# Sialylation of TLR2 initiates osteoclast fusion

**DOI:** 10.1038/s41413-022-00186-0

**Published:** 2022-03-02

**Authors:** Ce Dou, Gehua Zhen, Yang Dan, Mei Wan, Nathachit Limjunyawong, Xu Cao

**Affiliations:** 1 Department of Orthopedic Surgery, Institute of Cell Engineering and Department of Biomedical Engineering, Johns Hopkins University School of Medicine, Baltimore, MD USA; 2 The Solomon H. Snyder Department of Neuroscience, Johns Hopkins University School of Medicine, Baltimore, MD USA

**Keywords:** Bone, Metabolism

## Abstract

The molecular control of osteoclast formation is still not clearly elucidated. Here, we show that a process of cell recognition mediated by Siglec15-TLR2 binding is indispensable and occurs prior to cell fusion in RANKL-mediated osteoclastogenesis. Siglec15 has been shown to regulate osteoclastic bone resorption. However, the receptor for Siglec15 has not been identified, and the signaling mechanism involving Siglec15 in osteoclast function remains unclear. We found that Siglec15 bound sialylated TLR2 as its receptor and that the binding of sialylated TLR2 to Siglec15 in macrophages committed to the osteoclast-lineage initiated cell fusion for osteoclast formation, in which sialic acid was transferred by the sialyltransferase ST3Gal1. Interestingly, the expression of Siglec15 in macrophages was activated by M-CSF, whereas ST3Gal1 expression was induced by RANKL. Both Siglec15-specific deletion in macrophages and intrafemoral injection of sialidase abrogated cell recognition and reduced subsequent cell fusion for the formation of osteoclasts, resulting in increased bone formation in mice. Thus, our results reveal that cell recognition mediated by the binding of sialylated TLR2 to Siglec15 initiates cell fusion for osteoclast formation.

## Introduction

Macrophage/monocyte survival and proliferation are maintained by macrophage colony stimulating factor (M-CSF), and receptor activator of NF-kappaB ligand (RANKL) further promotes commitment to the osteoclast lineage as tartrate-resistant acid phosphatase-positive (Trap^+^) mononuclear cells, which are also known as preosteoclasts.^1,2^ In an appropriate microenvironment, preosteoclasts subsequently undergo cell–cell fusion to form Trap^+^ multinuclear osteoclasts.^3,4^ In particular, alterations in osteoclast differentiation or activity can result in almost every major skeletal disorder, such as osteoporosis, skeletal degeneration and pain, arthritis, and Paget disease. Loss-of-function mutations in or deletion of M-CSF causes severe osteopetrosis in patients with no osteoclast-lineage cells. Mice with a nullizygous M-CSF deletion (*Csf1*^*op*^*/Csf1*^*op*^) also develop osteopetrosis.^5^ In patients with a RANKL mutation, macrophage-lineage cells are not able to commit to differentiation into osteoclasts. Similarly, RANKL-deficient (*Tnfsf11*^−/−^) mice have problems in osteoclast differentiation with an osteopetrosis phenotype.^6^ Both M-CSF and RANKL are needed for osteoclast formation, but neither of them can induce osteoclast formation alone. Together, M-CSF and RANKL effectively induce the expression of osteoclast lineage-specific genes and lead to the development of osteoclast maturation marked by cell fusion-mediated multinucleation.^1,3^ The requirement for cell fusion in osteoclast formation has been studied for decades, and numerous fusogenic molecules have been identified. However, the mechanism controlling the initiation of cell fusion during osteoclast formation remains unknown.

Toll-like receptors (TLRs) are critical in the innate immune response and function by recognizing pathogen-associated molecular patterns with myeloid differentiation primary response protein 88 (MyD88) as a main adaptor.^7^ Trap^+^ macrophages/mononuclear cells fuse with their own kind instead of a similar cell type and are also dependent on a self-recognition mechanism. Interestingly, TLRs have an inhibitory effect on osteoclast differentiation upon agonist stimulation.^8^ TLR signaling is regulated by various mechanisms, such as ectodomain modification of N-linked glycans orchestrating TLR signaling capacities. The removal of sialyl residues from TLR glycosylation sites after neuraminidase treatment is enhanced following agonist stimulation.^9,10^ This suggests that TLR function can potentially be blocked by sialylation to ensure the normal progress of osteoclast differentiation.

Sialylation is a process mediated by sialyltransferases (STs), which catalyze the transfer of a sialic acid (SA) moiety to various acceptors in different linkages. SAs compose a family of nine-carbon acidic monosaccharides on N- and O-linked glycans and are attached to galactose or N-acetylgalactosamine units via α2,3- or α2,6-linkages.^11^ Sialylated glycoconjugates are involved in various cellular events, such as cell adhesion, hematopoietic stem cell fate determination, and viral fusion.^12–14^ SAs are specifically bound by SA-binding immunoglobulin-type lectins (Siglecs) that are primarily found on the surface of immune-related cells. Increases in SA have been widely implicated in different skeletal diseases,^15^ and blockade of SA has been shown to be effective in suppressing tumor growth by enhancing CD8^+^ T-cell activation.^16,17^

In this study, we sought to characterize the molecular mechanism of cell recognition that initiates osteoclast fusion. We found that TLR2 functioned as a Siglec15 receptor and that sialylation of TLR2 enabled osteoclast precursors to recognize themselves from nonself cells. Removal of sialic acid from TLR2 disabled cell recognition mediated by Siglec15 and inhibited consequential osteoclast fusion. Our finding of osteoclast recognition signaling is helpful in understanding the pathogenesis of different skeletal disorders and bone loss during aging.

## Results

### Siglec15 deficiency in macrophage-lineage cells reduced osteoclast fusion and bone resorption

**Fig. 1:**
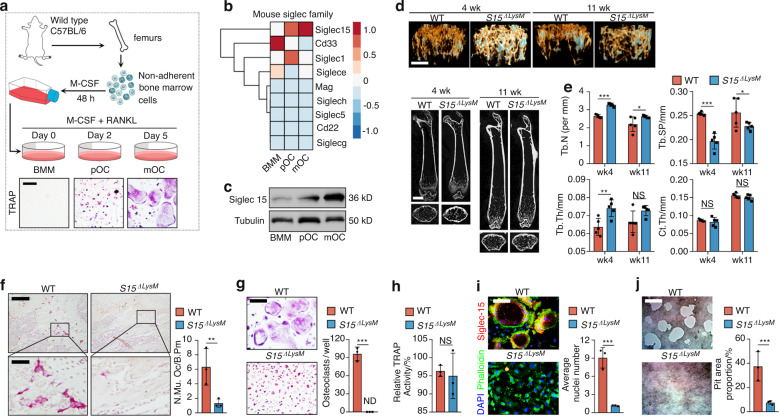
Siglec15 deficiency in macrophage-lineage cells leads to cell fusion failure and a decrease in bone volume. **a** Illustration of the preparation for Trap^+^ pOC and mOC differentiation. **b** Hierarchical clustering heatmap showing the expression of the mouse Siglec family in BMMs, pOCs, and mOCs detected and analyzed by RNA-seq. **c** Western blot analysis of Siglec15 in BMMs, pOCs and mOCs. **d** Representative μCT images of femurs from *Siglec15*^fl/fl^ and *Siglec15*^ΔLysM^ mice: 3D trabecular reconstruction (top) and 2D cross-sectional view (bottom). The bar represents 4 mm. **e** Quantitative μCT analysis of trabecular number (Tb.N), trabecular thickness (Tb.Th), trabecular separation (Tb.Sp), and cortical thickness (Ct.Th); *n* = 5. **f** TRAP staining of distal *Siglec15*^fl/fl^ and *Siglec15*^ΔLysM^ femur sections and quantification of the multinuclear osteoclast number; the bar represents 200 μm. TRAP staining with quantification of the osteoclast number per well (**g**) and relative TRAP activity (**h**) of BMMs isolated from *Siglec15*^fl/fl^ and *Siglec15*^ΔLysM^ mice. **i** Immunostaining of *Siglec15*^fl/fl^ (WT) and *Siglec15*^ΔLysM^ osteoclasts with phalloidin and anti-Siglec15; the average number of nuclei was quantified. **j** Bone resorption assay of *Siglec15*^fl/fl^ (WT) and *Siglec15*^ΔLysM^ osteoclasts quantified by pit area formation, *n* = 3. The bar represents 100 μm. Data represent the mean ± SD, and statistically significant differences are indicated as *P < 0.05; **P < 0.01; ***P < 0.001

Whole bone marrow cells were isolated from C57BL/6 mouse hind limbs and stimulated with M-CSF (50 ng·mL^−1^) for 48 h to acquire bone marrow macrophages (BMMs). BMMs were primed with RANKL to acquire Trap^+^ macrophages, which were then committed to the osteoclast lineage as preosteoclasts (pOCs) or mature osteoclasts (mOCs) (Fig. 1a). Next, we performed an unbiased global transcriptomic comparison of BMMs, pOCs, and mOCs by RNA sequencing (RNA-seq). The expression profile of the total mouse Siglec family was clustered, showing that Siglec15, which is conserved in most mammals, including humans, was the only continuously upregulated Siglec during osteoclast differentiation (Fig. 1b). The upregulation of Siglec15 at the protein level was validated by western blotting (Fig. 1c). To study the role of osteoclast-associated Siglec15 in vivo, we crossed *Siglec15*^fl/fl^ mice with the LysM-Cre strain to produce Siglec15 deletion in macrophages/granulocytes (*Siglec15*^ΔLysM^).^18^ Siglec15 is found on only macrophages, not granulocytes; therefore, this strain could be used as a specific tool to investigate the function of Siglec15 in BMMs, pOCs, and mOCs. We then used μCT to evaluate bone volume changes in young (4-week-old) and adult (11-week-old) *Siglec15*^ΔLysM^ mice relative to those in *Siglec15*^fl/fl^ littermates (Fig. 1d). Consistent with previous studies using global Siglec15 knockout mice,^19,20^ our results suggested an increase in trabecular bone volume in young and adult *Siglec15*^ΔLysM^ mice, marked by significant increases in trabecular number (Tb. N) and trabecular thickness (Tb. Th), as well as a significant decrease in trabecular separation (Tb. Sp). No significant change in cortical thickness (Ct. Th) was observed (Fig. 1e). TRAP staining of distal femur sections revealed that in young and adult *Siglec15*^ΔLysM^ mice, the multinucleated osteoclast number was much lower, the cell size was small, and cells did not spread well on the bone surface (Figs. 1f, S1). Immunofluorescence staining for Siglec15 and TRAP confirmed that Siglec15 and TRAP levels were robustly decreased in young and adult *Siglec15*^ΔLysM^ mouse distal femurs (Fig. S2).

To further investigate the role of Siglec15 in osteoclast formation, we isolated and cultured BMMs from *Siglec15*^ΔLysM^ mice and induced the cells with M-CSF and RANKL. TRAP staining results showed that on Day 5, large multinucleated osteoclasts were formed in the wild-type (WT) group but were not detected in the *Siglec15*^ΔLysM^ group (Fig. 1g). However, the relative TRAP activity results showed no obvious difference between the two groups (Fig. 1h). Immunofluorescence staining with anti-Siglec15 and phalloidin showed that Siglec15-deficient BMMs failed to form multinucleated osteoclasts with a complete actin ring (Fig. 1i). A pit formation assay showed a robust decrease in the bone resorption activity of Siglec15-deficient osteoclasts, marked by a significantly decreased pit area proportion (Fig. 1j).

### Sialylated TLR2 binds to Siglec15 as a Siglec15 receptor

**Fig. 2:**
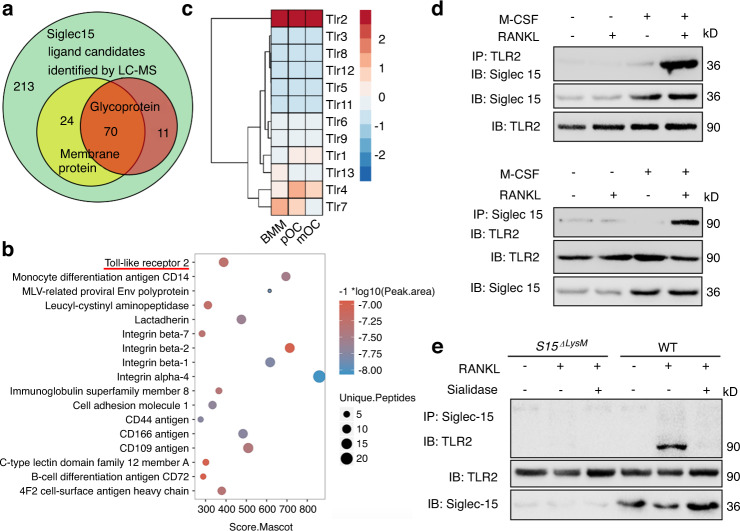
Sialylated TLR2 binds to Siglec15 as a Siglec15 receptor. **a** Identification of 318 potential Siglec15 binding proteins using the proximity labeling method followed by LC–MS/MS. The number of membrane proteins and glycoproteins is shown. **b** Most highly enriched glyco-membrane protein evaluated by the Mascot score, peak area and unique peptides. **c** Hierarchical clustering heatmap showing the expression of mouse TLR family members in BMMs, pOCs and mOCs detected by RNA-seq. **d** Immunoblotting (IB) for Siglec15 in whole-cell lysates or protein complexes immunoprecipitated (IP) with TLR2 and IB for TLR2 in whole-cell lysates or protein complexes IP with Siglec15 in the indicated groups. **e** IB for TLR2 in whole-cell lysates or protein complexes IP with Siglec15 in *Siglec15*^ΔLysM^ or *Siglec15*^fl/fl^ (WT) BMMs to test the effects of sialidase

To determine the ligands of Siglec15, we adopted a liquid chromatography–mass spectrometry (LC–MS) dataset (ProteomeXchange Consortium, PXD006359) identifying Siglec15-interacting proteins using proximity labeling methods.^21^ The results identified 318 proteins as potential candidate Siglec15 ligands. The candidate number was decreased to 70 after narrowing the scope to membrane glycoproteins (Fig. 2a). TLR2 was screened out with the highest overall score for unique peptides, peak area, and Mascot score (Fig. 2b). Using bulk RNA-seq, we also found that among its family members, TLR2 was consistently expressed at a high level during osteoclastogenesis (Fig. 2c). The interaction of Siglec15 and TLR2 in WT BMMs induced to become osteoclasts was verified by co-immunoprecipitation (co-IP) (Fig. 2d). The Siglec15-TLR2 interaction was not detected in BMMs from *Siglec15*^ΔLysM^ mice. Furthermore, sialidase treatment disrupted the formation of the Siglec15-TLR2 complex in WT osteoclasts, suggesting that sialylation of TLR2 is essential for Siglec15-TLR2 complex formation (Fig. 2e). To test the effects of sialidase disruption on the Siglec15-TLR2 interaction in osteoclast differentiation, we treated WT BMMs with sialidase at different time points. Sialidase treatment did not affect the formation of Trap^+^ pOCs or the bone resorption activity of mOCs; however, sialidase treatment did block the fusion of pOCs into multinucleated mOCs (Fig. S3).

### RANKL-induced *ST3Gal1* transcription by activating the binding of FOS to its promoter

**Fig. 3:**
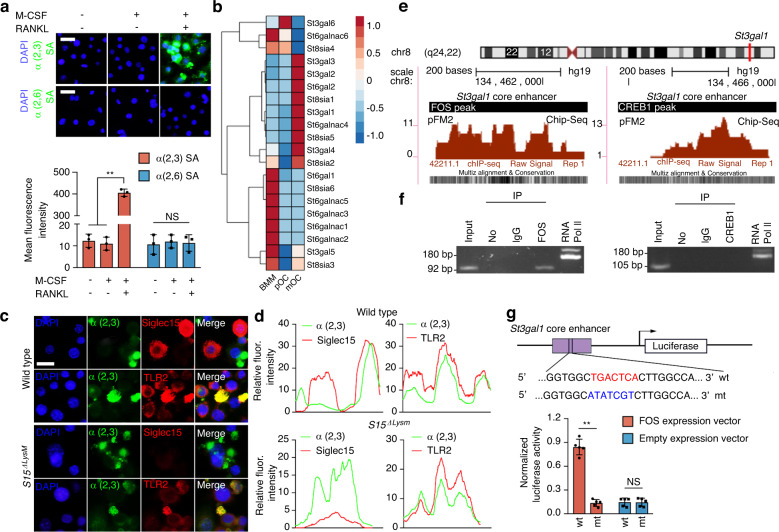
RANKL-induced *ST3Gal1* transcription by activating the binding of FOS to its promoter. **a** Immunostaining of α2,3 and α2,6 sialic acids (SAs) in WT BMMs treated as indicated. Quantification of the fluorescence intensity. **b** Hierarchical clustering heatmap showing the expression of SA transferase family members in BMMs, pOCs and mOCs detected and analyzed by RNA-seq. **c** Immunostaining of α2,3 SA with TLR2 or Siglec15 in *Siglec15*^fl/fl^ (WT) and *Siglec15*^ΔLysM^ BMMs. **d** Colocalization of α2,3 SA with TLR2 or Siglec15. **e** FOS and CREB1 binding sites in the *St3gal1* promoter core enhancer. **f** ChIP assay validation of FOS and CREB binding activities. **g** Site-directed mutagenesis of the FOS binding sites in the *St3gal1* core enhancer. Quantification of the normalized luciferase activity of a luciferase gene reporter assay performed with BMMs transfected with a c-Fos expression vector, *n* = 5. Data represent the mean ± SD, and statistically significant differences are indicated as **P < 0.01

Because TLR2 has 4 N-linked glycosylation sites,^9^ we tested α2,3 and α2,6 N-linked SA modifications, the two most common sialylation patterns in mammalian cells, to investigate the molecular mechanism of sialylation. We found that RANKL, not M-CSF, robustly induced α2,3 sialylation of TLR2 in WT BMMs (Fig. 3a). To understand why α2,3 SA was specifically modified, we examined the expression profile of each member of the entire sialyltransferase family. The RNA-seq data for osteoclasts at different stages showed that among the STs that are expressed in skeletal tissue, *ST3Gal1*, which encodes an enzyme catalyzing α2,3 sialylation, was significantly upregulated, whereas *ST6Gal1*, which encodes an enzyme catalyzing α2,6 sialylation, was downregulated (Fig. 3b). Moreover, α2,3 SA was immunocolocalized with TLR2 but not with Siglec15 in WT and *Siglec15*^ΔLysM^ BMMs after RANKL stimulation for 24 h (Fig. 3c, d).

To test the signaling activity of α2,3 sialylation, we removed the α2,3 SAs on TLR2 induced by RANKL by adding sialidase to a BMM culture. ELISA showed that the peptidoglycan-induced expression of tumor necrosis factor (TNF)-α and interleukin (IL)-6 by BMMs mediated through TLR2 activation was inhibited by RANKL and that the addition of sialidase restored TLR2 activity and the TNF-α and IL-6 levels; however, lipopolysaccharide (LPS) stimulation was not affected by RANKL or sialidase (Fig. S4). To examine the transcriptional mechanism of ST3Gal1 activation by RANKL, we analyzed the 5′ promoter sequence of *ST3Gal1* and recognized binding sites for CREB1 and FOS (downstream transcription factors of RANKL signaling) in the core enhancer (Fig. 3e). A chromatin immunoprecipitation (ChIP)-PCR assay showed that FOS but not CREB1 bound to the core enhancer of *ST3Gal1* (Fig. 3f). To confirm the functional activity of the FOS site, we constructed an enhancer reporter with the *ST3Gal1* enhancer and mutant FOS binding sites. Transcriptional activity analysis showed that the FOS recognition sequence TGACTCA was responsible for the FOS-dependent activation of ST3Gal1 (Fig. 3g). To validate RANKL-induced sialylation in vivo, we crossed *RANKL*^fl/fl^ mice with the Dmp1-Cre strain to generate osteocyte-specific RANKL knockout mice (*RANKL*^ΔDmp1^). Indeed, immunohistochemical staining showed that the expression of ST3Gal1 in the distal femur of *RANKL*^ΔDmp1^ mice was significantly decreased relative to that in *RANKL*^fl/fl^ mice (Fig. S5). Furthermore, α2,3 SA levels were significantly downregulated in *RANKL*^ΔDmp1^ mice relative to *RANKL*^fl/fl^ mice (Fig. S6A). Additionally, the Siglec15-TLR2 colocalization that was observed in *RANKL*^fl/fl^ mice disappeared in *RANKL*^ΔDmp1^ mice (Fig. S6B). An in vitro study confirmed that *St3gal1* siRNA transfection disturbed osteoclast fusion, as indicated by a robust reduction in the number of osteoclasts (Fig. S7).

### Siglec15 expression in macrophages/monocytes is induced by M-CSF via MEK-ERK-MYC signaling

**Fig. 4:**
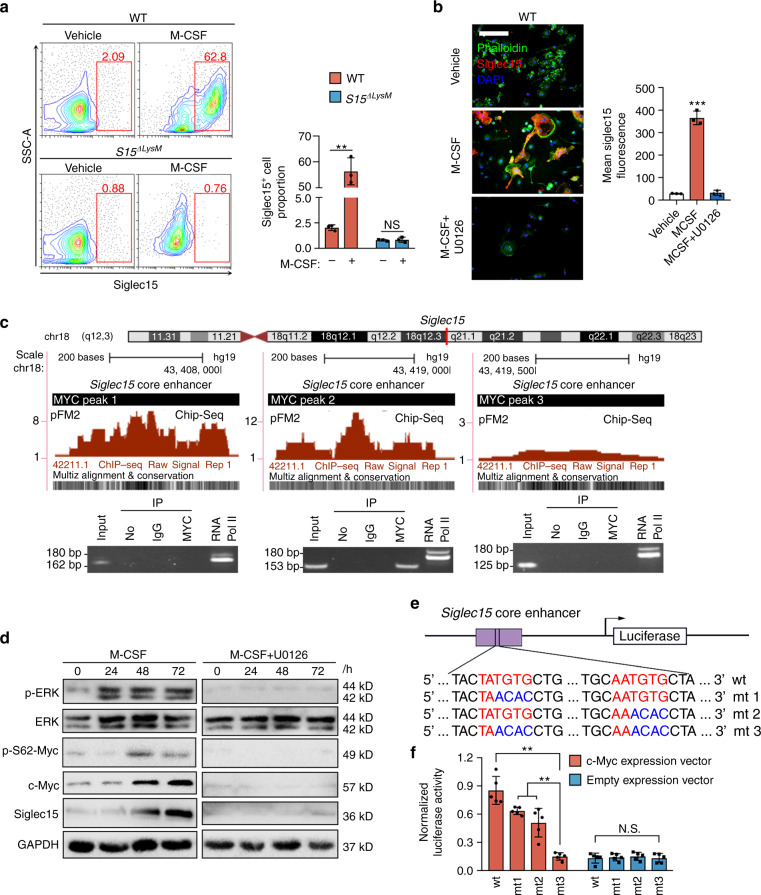
Siglec15 expression in macrophages/monocytes is induced by M-CSF via MEK-ERK-MYC signaling. **a** Flow cytometric analysis of *Siglec15*^fl/fl^ (WT) and *Siglec15*^ΔLysM^ BMMs treated with M-CSF or vehicle for 3 days, with quantification of the Siglec15^+^ cell proportion. **b** Immunostaining of BMMs treated with M-CSF, M-CSF + U0126 or vehicle with phalloidin and anti-Siglec15, with quantification of Siglec15^+^ cells; the bar represents 50 μm. **c** Three MYC binding sites in the *Siglec15* promoter core enhancer region and chromatin immunoprecipitation (ChIP) assay validation of the MYC binding sites. **d** Western blot analysis of ERK, MYC phosphorylation, and Siglec15 in BMMs treated with M-CSF, M-CSF + U0126 or vehicle at 0, 24 h, 48 h, and 72 h. **e** Site-directed mutagenesis of the MYC binding sites in the *Siglec15* core enhancer. **f** Quantification of the normalized luciferase activity of a luciferase gene reporter assay performed with BMMs transfected with a c-Myc expression vector, *n* = 5. Data represent the mean ± SD, and statistically significant differences are indicated as **P < 0.01; ***P < 0.001

Using flow cytometry, we confirmed that in vitro M-CSF stimulation of whole bone marrow cells significantly increased the proportion of Siglec15^+^ BMMs from *Siglec15*^fl/fl^ mice, whereas cells from *Siglec15*^ΔLysM^ mice showed no obvious changes (Fig. 4a). To explore the molecular mechanism of the M-CSF-mediated activation of Siglec15, we used U0126, a selective mitogen-activated protein kinase kinase inhibitor of MEK1 and MEK2. BMMs treated with U0126 showed decreases in osteoclastogenesis and the cell survival rate (Fig. S8). Siglec15 upregulation by M-CSF was also abrogated by U0126 treatment (Fig. 4b). To understand *Siglec15* transcription, we analyzed the core enhancer of *Siglec15* and found three binding sites for MYC with high species conservation. A ChIP assay was used to detect the interaction of MYC with the 3 deoxyribonucleic acid binding sites. Electrophoresis of the PCR amplification products showed that MYC bound with a 310-bp region in the core enhancer of *Siglec15* (Fig. 4c). Western blot results confirmed that M-CSF-mediated extracellular signal-regulated kinase (ERK) activation resulted in the phosphorylation of Ser62 of MYC, thus stabilizing MYC to further activate Siglec15 expression. U0126 treatment blocked this cascade from inhibiting ERK activation (Fig. 4d). ChIP-PCR results showed that GM-CSF could not induce the binding of MYC with the core enhancer of *Siglec15* (Fig. S9). MYC recognizes and binds with the sequence CACGTG or CATGTG in E-box domains.^22^ In the 310-bp binding region, we recognized the sequences TATGTG and AATGTG, which share 80% similarity with reported binding bases. To validate the binding of these MYC recognition sequences, we constructed an enhancer reporter with this *Siglec15* enhancer, and site-directed mutagenesis was used to generate 3 mutants to eliminate MYC recognition (Fig. 4e). Enhancer analysis revealed that these MYC binding sequences were responsible for the MYC-dependent activation of Siglec15 (Fig. 4f). Thus, M-CSF activates Siglec15 expression via the MEK-ERK-MYC cascade.

### Binding of Siglec15 and TLR2 activates both of their downstream signaling pathways

**Fig. 5:**
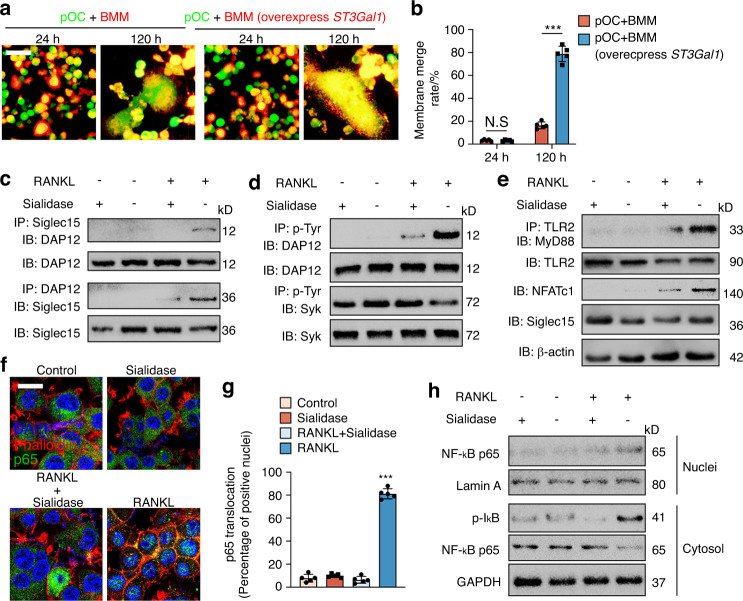
Binding of Siglec15 and TLR2 activates both of their downstream signaling pathways. **a** Trap^+^ mononuclear cells were marked with CellTracker Green, and BMMs with or without St3gal1 overexpression were marked with the cell label DiI. The cells were then cocultured for 24 or 72 h before observation using a fluorescence microscope. **b** Quantification of the membrane merge rate in (**a**). **c** Immunoblotting (IB) for DAP12 in whole-cell lysates or protein complexes immunoprecipitated (IP) with Siglec15 and IB for Siglec15 in whole-cell lysates or protein complexes IP with DAP12 in the indicated groups. **d** IB for DAP12 and Syk in whole-cell lysates or protein complexes IP with p-Tyr in the indicated groups. **e** IB for MyD88 in protein complexes IP with TLR2 in the indicated groups. IB for TLR2, NFATc1, and Siglec15 in whole-cell lysates. **f** Immunostaining with phalloidin (red) and anti-p65 (green) in the indicated groups to observe p65 nuclear translocation. Scale bar = 20 µm. **g** Quantification of the number and percentage of p65-positive nuclei. **h** Western blot analysis of p‐IκBα and p65 in the cytosol and p65 in the nucleus in the indicated groups. Data represent the mean ± SD, and statistically significant differences are indicated as ***P < 0.001

Siglec15 deficiency led to smaller OCs that were still TRAP^+^, so we further tested the gene expression of the osteoclastogenesis master regulator *Nfat2* (NFATc1*)*, osteoclastic marker *Acp5* (TRAP), *ctsk*, and the fusogenic genes *ocstamp* and *Tm7sf4* (DC-STAMP) in WT and *Siglec15*^ΔLysM^ BMMs using qPCR. The results showed that upon M-CSF + RANKL stimulation, the expression of *Nfat2*, *Acp5*, *ctsk*, *oc-stamp*, and *Tm7sf4* was not affected by Siglec15 deficiency (Fig. S10). These results suggest that the process of cell recognition mediated by Siglec15 binding to its receptor sialylated TLR2 occurs prior to cell fusion. We assumed that the cell recognition patterned by Siglec15-TLR2 binding enables Trap^+^ macrophages to distinguish themselves from nonself cells and serves as a prerequisite for cell fusion. For validation, Trap^+^ mononuclear cells were cocultured with BMMs, and no obvious fusion was detected; however, Trap^+^ mononuclear cells cocultured with BMMs overexpressing *ST3Gal1* showed a significantly higher membrane fusion rate with observable fused multinucleated osteoclasts (Fig. 5a, b), indicating that binding between Siglec15 and sialylated TLR2 initiates cell fusion. In particular, the cell recognition mediated by Siglec15 binding to its receptor sialylated TLR2 suggests activation of downstream signaling by both Siglec15 and sialylated TLR2. We therefore examined whether the binding between Siglec15 and sialylated TLR2 activates bidirectional signaling in Trap^+^ mononuclear cells during the initiation of cell fusion.

To examine whether Siglec15 downstream signaling is activated by binding to sialylated TLR2, co-IP of Siglec15 with DAP12, a key factor for cell fusion, was conducted in BMMs induced with RANKL, sialidase or both. RANKL-induced the association of Siglec15 with DAP12, while sialidase impaired the association (Fig. 5c). Moreover, tyrosine phosphorylation of DAP12 was detected in the co-IP experiment upon RANKL induction of Siglec15 clustering but was again impaired by sialidase treatment (Fig. 5d). We then tested whether TLR2 signaling is activated by binding to Siglec15. The recruitment of MyD88 to TLR2 was examined by co-IP. Binding of Siglec15 induced TLR2 association with MyD88 and resulted in upregulation of NFATc1. Removal of SA by sialidase reduced MyD88 recruitment and NFATc1 activation (Fig. 5e). Immunostaining further revealed that p65 nuclear translocation activated by RANKL was downregulated by sialidase, resulting in the sequestration of p65 in the cytoplasm (Fig. 5f, g). Western blot analysis of the subcellular distribution of p65 showed that RANKL-induced p65 nuclear translocation and cytosolic IκBα phosphorylation and that sialidase treatment dampened these effects (Fig. 5h). Cell–cell fusion is a fundamental cellular activity of macrophages, and multinucleation is a complex process that is likely involved in multiple signaling pathways. Activation of downstream signaling by both Siglec15 and sialylated TLR2 is involved in different cells simultaneously, and both signaling pathways could also occur in each cell, which could be the critical mechanism for cell fusion.

### Sialidase-mediated reductions in osteoclast formation and bone remodeling were abrogated in *Siglec15*^ΔLysM^ mice

**Fig. 6:**
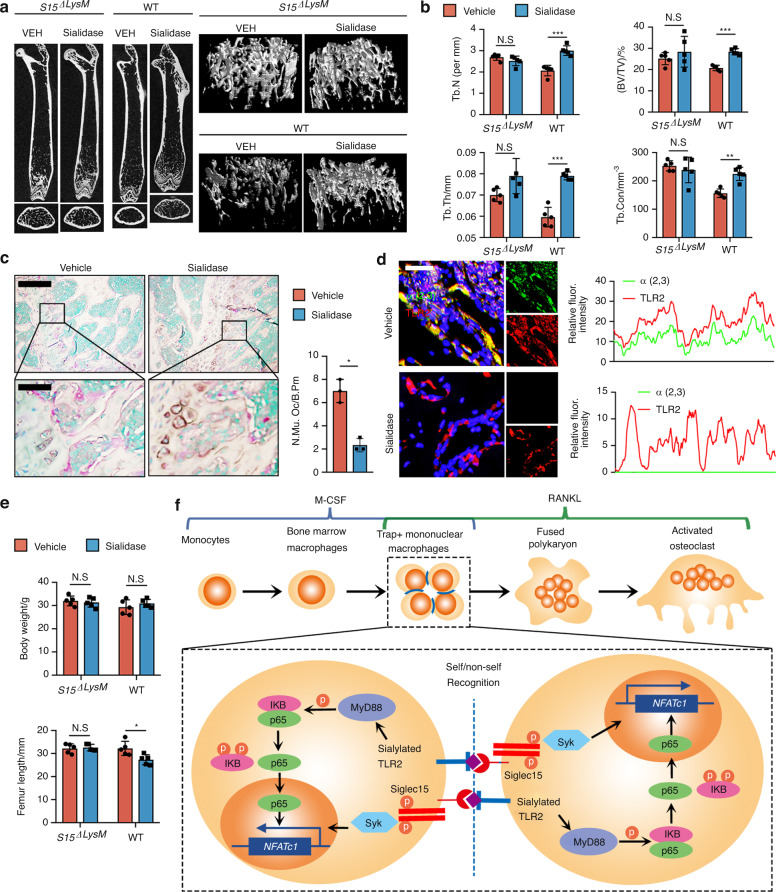
Sialidase-mediated reductions in osteoclast formation and bone remodeling were abrogated in *Siglec15*^ΔLysM^ mice. **a** Representative μCT cross-sectional femur images of *Siglec15*^fl/fl^ (WT) and *Siglec15*^ΔLysM^ mice intrafemorally injected with vehicle (VEH) or sialidase. The bar represents 4 mm. **b** Quantitative μCT analysis of trabecular number (Tb.N), trabecular thickness (Tb.Th), bone volume/tissue volume (BV/TV), and trabecular connectivity (Tb. Con); *n* = 5. **c** TRAP staining of distal femur sections from *Siglec15*^fl/fl^ (WT) mice intrafemorally injected with vehicle or sialidase and quantification of the multinuclear osteoclast number, *n* = 3. **d** Immunostaining of α2,3 SA and TLR2 in distal femur sections from *Siglec15*^fl/fl^ (WT) mice intrafemorally injected with vehicle or sialidase and colocalization analysis of α2,3 with TLR2. **e** Body weight and femur length of *Siglec15*^fl/fl^ (WT) and *Siglec15*^ΔLysM^ mice intrafemorally injected with vehicle or sialidase, *n* = 5. **f** Schematic diagram showing the self/nonself-recognition mediated by siglec15 binding with sialylated TLR2 before further cell fusion. Data represent the mean ± SD, and statistically significant differences are indicated as *P < 0.05; **P < 0.01; ***P < 0.001

To validate that sialylation of TLR2 originates with binding to Siglec15 for osteoclast fusion, we injected sialidase intrafemorally into *Siglec15*^ΔLysM^ mice and their *Siglec15*^fl/fl^ littermates. After 4 weeks of injections, the mice were euthanized, and the femurs were collected for μCT scanning and histological analysis (Fig. 6a). μCT analysis indicated that trabecular bone volume (TV/BV), Tb. Th, Tb. N, and trabecular connectivity (Tb. Con) were significantly increased in *Siglec15*^fl/fl^ mice injected with sialidase (Fig. 6b), indicating a decrease in osteoclast-induced bone remodeling. Indeed, TRAP staining of distal femur sections showed that the number and size of multinucleated osteoclasts in the sialidase-treated mice were decreased significantly relative to those in control mice (Fig. 6c). The number of osteoclast precursors on the bone surface was slightly higher but not significantly different in *Siglec15*^ΔLysM^ mice treated with sialidase (Fig. S11). Further coimmunofluorescence staining for α2,3 SA and TRAP revealed that sialylation was abrogated in mouse distal femurs injected with sialidase (Fig. 6d), and again, no changes were observed in *Siglec15*^ΔLysM^ mice. The femurs of *Siglec15*^fl/fl^ mice injected with sialidase were shorter than those of vehicle-injected mice, suggesting a delay in femur growth, whereas no difference was observed between *Siglec15*^ΔLysM^ mice injected with sialidase or vehicle (Fig. 6e). A recent report showed that osteoclasts are recycled via fission into daughter osteomorphs.^23^ By testing the expression of *Bpgm* and *Fbxo7* in the bone marrow of OPG:Fc-treated WT and *Siglec15*^ΔLysM^ mice, a potential downregulation of the osteomorph reservoir was observed in *Siglec15*^ΔLysM^ mice (Fig. S12), suggesting an inhibition of multinucleated osteoclast formation. Our results show that the sialylation of TLR2 initiates its interaction with Siglec15 to induce cell fusion for osteoclast formation, bone remodeling, and bone growth.

## Discussion

Many skeletal disorders, including skeletal metastasis, involve osteoclast fusion and subsequent aberrant osteoclast differentiation. To date, we have not been able to develop effective therapies for these skeletal diseases, partly due to the limited knowledge about the molecular mechanism of osteoclast fusion by Trap^+^ mononuclear cells. It is quite clear that two factors are essential in osteoclast differentiation: M-CSF is required for progenitor cell survival and proliferation,^24^ and RANKL is required for commitment to osteoclast-lineage differentiation.^3^ In this study, we found that the binding of Siglec15 to sialylated TLR2, as a cell recognition signal, initiates the fusion of preosteoclasts, discriminating self from nonself. Importantly, Siglec15 expression is activated by M-CSF, while TLR2 sialylation is induced by RANKL (Fig. S13), indicating that both M-CSF and RANKL are needed for the formation of the recognition signal for osteoclast fusion (Fig. 6f). Siglec15 has been reported to regulate osteoclast differentiation by associating with DNAX-activating protein 12 kD (DAP12) upon activation by unknown ligands.^19,25,26^ We now show that the α2,3 sialylated TLR2 interaction with Siglec15 activates downstream signaling by both membrane proteins for the fusion of preosteoclasts. A recent cell-based glycan array study also reported that α2,3 SA has a specific high affinity for Siglec15.^27^ Prior to fusion, preosteoclasts exhibit distinctly anabolic function, whereas fused multinucleated osteoclasts are catabolic in bone resorption. Trap^+^ mononuclear cells are maintained on the periosteal surface for cortical bone formation.^28,29^ Trap^+^ mononuclear cells have been observed in the aquatic vertebrate skeleton, where there is continuous growth with no osteoclastic bone resorption.^30,31^ The well-controlled regulation between mononuclear preosteoclasts and fused multinucleated osteoclasts balances bone anabolic and catabolic activity. Shifting the balance toward osteoclast formation leads to bone loss, such as postmenopausal osteoporosis and breast cancer metastasis to the bone.

We previously reported that preosteoclasts secrete platelet-derived growth factor (PDGF)-BB for type H blood vessel formation in coupling osteogenesis.^32^ Preosteoclasts control the coupling between osteogenesis and angiogenesis during bone remodeling or modeling. In postmenopausal osteoporosis, preosteoclasts undergo fusion to form osteoclasts much earlier with a relatively short lifespan.^33,34^ As a result, osteoclastic bone resorption is increased, whereas blood vessel formation supporting bone formation is decreased, and the net outcome is catabolic bone loss. Hence, the signaling mechanism that triggers the fusion of preosteoclasts is critical in maintaining bone homeostasis, and understanding this mechanism is essential to developing potential therapies for immedicable skeletal disorders. Interestingly, TLR activation in BMMs abolished their differentiation into osteoclasts.^8,35^ Our results showed that TLRs on BMMs were modified with α2,3 glycans, induced by RANKL, as a ligand for Siglec15. The recognition and binding between Siglec15 and sialylated TLR2 activate Siglec15-associated DAP12, which costimulates osteoclast differentiation^4^ and blocks TLR signaling to inhibit osteoclast differentiation. It has been shown that cell-surface glycoconjugate sialylation is needed in osteoclast maturation.^36^ Importantly, we demonstrate that inhibition of sialylation blocks the TLR signaling needed for osteoclast fusion and differentiation. The binding of TLRs to Siglec15 may not be limited to TLR2, as interactions between TLRs and Siglec family members have been broadly detected.^37^ For example, it has been reported that Siglec-E binding with TLR4 negatively regulates its activation^38^ and that TLR-activated SOCS1 and SOCS3 expression is reduced in Siglec2-deficient B cells.^39^

Siglec15 suppresses T-cell activation, and an antibody against Siglec15 has shown notable potential in cancer immunotherapy. In the tumor microenvironment, Siglec15-expressing cancer cells and tumor-associated macrophages facilitate tumor growth and metastasis by suppressing T-cell activation.^18,40^ Siglec15 knockout in macrophage-lineage cells also abrogates osteoclast formation, but the signaling mechanisms are not known, as the receptor for Siglec15 has not been identified. We found that sialylated TLR2 binds to Siglec15 as a Siglec15 receptor. Naïve CD8^+^ T cells have significantly higher cell-surface sialylation levels than activated T cells, and the inhibitory effects of Siglec15 on T-cell activation are dependent on T-cell-surface SAs. The binding of Siglec15 or sialylation of TLRs alone could block the activation of CD8^+^ T cells. TLR2 and TLR4 are expressed in both T cells and macrophages and have regulatory roles involved in the effector functions and activation of these cells.^41–43^ Sialylation of TLRs potentially blocks the activation of T cells and macrophages by their agonizts through binding to Siglect15. During osteoclastogenesis, Siglec15 and sialylated TLR2 function as a self/nonself-recognition switch, and this binding activates both of their downstream signaling pathways to initiate cell fusion by Trap^+^ macrophages/monocytes.

Autoimmune diseases are often associated with imbalanced bone remodeling resulting in increased bone resorption, such as rheumatoid arthritis (RA) and systemic lupus erythematosus (SLE) arthritis. In RA, excessive RANKL is produced by synovial fibroblasts stimulated by T_H_17 cell-derived IL-17.^44^ In SLE patients, the soluble RANKL level is significantly increased in the serum.^45^ The upregulated RANKL in these autoimmune diseases accelerates the fusion of preosteoclasts. As a result, the lifespan of preosteoclasts is shortened with an increase in osteoclast maturation for bone degradation, which explains most autoimmune-related bone disorders with bone destruction. Indeed, serum sialic acid levels were shown to be increased in RA patients, with potential for use as biomarkers for RA severity prediction in clinical practice.^46,47^ In both SLE and RA patients, the ST3Gal1/Neu3 ratio was found to be positively correlated with disease activity.^48^ Similarly, in cancer, aberrant sialylation is highly associated with tumor metastasis, including bone invasion.^49^ Prostate cancer has the highest rate of spread to the bone,^50^ and an elevated serum SA level is an independent predictor of bone metastases in this disease.^51^ A metabolomic study also showed that SA plays a key role in breast cancer metastasis.^52^ Thus, our identification of the control signal for osteoclast recognition and fusion provides potential therapeutic targets for skeletal disorders associated with aberrant osteoclast activity.

## Methods

### Mice and treatment

WT C57BL/6J, LysM-Cre, Dmp1-Cre, and *RANKL*^fl/fl^ mouse strains (referred to as “WT”) were purchased from the Jackson Laboratory (Ellsworth, ME, USA). *Siglec-15*^fl/fl^ mice were obtained from Dr. L.C. at Yale University. A Siglec-15 conditional knockout mouse strain was generated by crossing *Siglec-15*^fl/fl^ mice with LysM-Cre mice (referred to as *Siglec15*^ΔLysM^).^18^ A RANKL conditional knockout mouse strain was generated by crossing *RANKL*^fl/fl^ mice with Dmp1-Cre mice (referred to as *RANKL*^ΔDmp1^).^53^ For time-course animal studies, WT littermates were used as controls, and male mice were euthanized with carbon dioxide asphyxiation for further analysis (10 to 12 per group). For sialidase injection, mice were anesthetized by intraperitoneal injection of ketamine (100 mg·kg^−1^) and xylazine (10 mg·kg^−1^). SialEXO 23 α2,3 specific sialidase (Genovis Inc, MA, USA) was prepared as 5 units preincubated in 20 mmol·L^−1^ Tris pH 7.5 at 37 °C for 1 h and then injected intrafemorally. All the mice used in this study were maintained at the Johns Hopkins University School of Medicine animal facility. The animal study protocols were approved by the Animal Care and Use Committee of Johns Hopkins University.

### RNA sequencing

Bulk RNA-seq was used to screen the gene expression profiles of the mouse siglec family and TLR family. The detailed steps were described in a previous report.^54^ In brief, total RNA was isolated from BMMs and osteoclasts at different stages. After an initial quality check and purification, the transcripts were fragmented and converted into cDNA for library creation. An Illumina NovaSeq 6000 platform was used for sequencing.

### μCT analysis

Male mice on different genetic backgrounds were used for analysis of bone phenotype. Carbon dioxide asphyxiation was used for mouse euthanasia. For μCT scanning, mouse femurs and tibias were dissected and fixed with paraformaldehyde for at least 24 h. A Bruker micro-CT Skyscan 1172 (Kontich, Belgium) system was used for scanning. The detailed scanning information, including isotropic voxel size, X-ray tube voltage, intensity, and exposure time, were described in our previous studies.^55^ In brief, 3D reconstruction of the region of interest in the mouse femur/tibia was realized by Nrecon (Kontich, Belgium). Contoured 2D images were analyzed using CTvox (Kontich, Belgium). Data analysis was performed using a CT analyzer (Kontich, Belgium).

### In vitro osteoclast formation assay

For TRAP staining, cells were first fixed using paraformaldehyde at 37 °C for 5 min before staining with a TRAP solution. The staining procedures were strictly performed according to the instructions of the manufacturer (Sigma-Aldrich, St. Louis, MO, USA) before light microscopy observation. For sialidase treatment, 1 U·mL^−1^ SialEXO 23 α2,3 sialidase and 10 μmol·L^−1^ U0126 were used for cell culture. The procedures were reported in detail in our previous studies.^56,57^ In brief, cells were washed, fixed, and permeabilized, followed by blocking. A primary antibody against vinculin (1:1 000) was incubated for 12 h at 4 °C. DAPI (1:2 000) was used for nuclear counterstaining. For the bone resorption assay, cells were incubated on bovine bone slices and then placed in 48-well plates for osteoclastic stimulation. The cells were removed from the slice surface with a bleach solution for further observation of pit formation.

### IHC and IF

Mouse bone specimens were first fixed and then decalcified using 10% EDTA (Sigma-Aldrich, St. Louis, MO, USA) for 14 days with constant shaking. For the histological assays, the detailed protocols were reported in a previous study.^58^ In short, the samples were then dehydrated and embedded in optimal cutting temperature compound (Sakura Finetek, Torrance, CA, USA) or in paraffin. Four-μm-thick coronal-oriented femur sections were prepared for TRAP staining. Forty-μm-thick coronal-oriented femur sections were prepared for IF staining. The detailed protocols were described in a previous study.^58^ Briefly, the sections were incubated with primary antibodies against mouse TLR2 (Santa Cruz Biotechnology, sc-21759, 1:200), Siglec15 (PA5-48221, Thermo Fisher Scientific, 1:100), ST3GAL1 (PA5-21721, Thermo Fisher Scientific, 1:50), and TRAP (Abcam, ab191406, 1:100) for 12 h at 4 °C. For sialic acid detection, biotinylated Maackia Amurensis Lectin II (MAL II) (Vector Laboratories, CA, USA) was used to label the α2,3 linkage, and biotinylated Sambucus Nigra Lectin (SNA) (Vector Laboratories, CA, USA) was used to label the α2,6 linkage. Fluorescein-conjugated streptavidin (Vector Laboratories, CA, USA) was used for the addition of a fluorescent label to biotinylated sialic acid conjugates. A Zeiss LSM 780 confocal microscope and an Olympus BX51 microscope were used for image capture.

### ChIP-PCR assay

For the ChIP assay, primary BMMs were cultured with M-CSF or GM-CSF stimulation for 48 h to detect *Siglec15* core enhancer DNA binding. M-CSF-primed cells were induced with RANKL for another 72 h to detect *St3gal1* core enhancer DNA binding. Afterward, the cells were cross-linked using 1% formaldehyde and lysed. DNA fragmentation was then achieved by enzymatic digestion with micrococcal nuclease (MNase). After digestion, 10% of the sample was preserved as the total input aliquot for further use. The remaining supernatant was then incubated with a ChIP-grade primary antibody against mouse p-CREB (Abcam, ab32096, 10 μg), c-FOS (Abcam, ab27793, 10 μg), or c-MYC (Abcam, ab9132, 10 μg). An anti-RNA Polymerase II antibody was used as the positive IP control, and normal rabbit IgG was used as the negative IP control. The supernatant was incubated overnight at 4 °C with mixing before immunoprecipitation using ChIP-grade Protein A/G Magnetic Beads following the suggestions of a ChIP kit (26157, Thermo Fisher Scientific). After elution, the DNA was then purified and recovered according to the manufacturer’s instructions, and PCR detection was performed. The PCR primers used to detect MYC binding were as follows: Site #1, forward: 5′-TGCGGTGACTGATATACGCA-3′, and reverse: 5′-ACCATTTTCTCTTGCTCGCG-3’; Site #2, forward: 5′-GGTCACGGCTACCAGGTG-3′, and reverse: 5′-GTGGAAGCGGAACAGGTAGA-3’; and Site #3, forward: 5′-TGCGGTGACTGATATACGCA-3′, and reverse: 5′-ACCATTTTCTCTTGCTCGCG-3’. The PCR primers used to detect FOS binding were as follows: forward: 5′-GCCCAGTGACGTAGGAAGTC-3′, and reverse: 5′-GTCGCGGTTGGAGTAGTAGG-3’. The PCR primers used to detect CREB binding were as follows: forward: 5′-CAGCGAGCTGTGCCAGAC-3′, and reverse: 5′-AACTCCACGCGGCAGAAGTA-3’. ChIP positive control primers (GAPDH promoter) were provided in the kit (26157, Thermo Fisher Scientific). The PCR program comprised 40 cycles of 95 °C for 20 s, 62 °C for 60 s, and 72 °C for 30 s. For visualization and gel staining, 5 μL of PCR products was added to 1.5% agarose gels (Millipore Sigma, MO).

### Bioinformatics

The prediction of gene core enhancer transcription factor binding was achieved with the Encyclopedia of DNA Elements project, and ChIP-seq data were supported by the UCSC genome browser. The determination of Siglec15 ligand candidates was performed using a LC–MS dataset (ProteomeXchange Consortium, PXD006359), and the results were visualized using the R program. All raw data and processed bulk RNA-seq data were acquired from the GEO database (GSE133515).

### Co-immunoprecipitation and a luciferase reporter assay

For the co-IP assays, cells were first lysed in the presence of protease inhibitors using IP buffer. The lysates were then immunoprecipitated using primary antibodies against mouse TLR2 (Santa Cruz Biotechnology, sc-21759) and Siglec15 (PA5-48221, Thermo Fisher Scientific), followed by absorption on Protein A/G as suggested by the manufacturer (26149, Thermo Fisher Scientific). SDS–PAGE was then used to separate the immunoprecipitates before transfer to a nitrocellulose membrane for immunoblotting procedures. The membranes were incubated with primary antibodies against mouse p-ERK (44-680G, Thermo Fisher Scientific, 1:1 000), ERK (13-6200, Thermo Fisher Scientific, 1:1 000), p-S62-Myc (ab51156, Abcam, 1:1 000), c-Myc (ab32072, Abcam, 1:1 000), TLR2 (Santa Cruz Biotechnology, sc-21759, 1:1 000), and Siglec15 (PA5-48221, Thermo Fisher Scientific, 1:1 000) for 12 h at 4 °C, followed by a 1-h incubation with a secondary antibody (1:1 000). The Dual-Luciferase Reporter Assay System (Promega, Madison, WI, USA) was used to detect luciferase activity. Enhancer assays were performed with adjustment for transfection efficiency differences.^59^ Reporter cotransfection assays were performed with a reporter-to-activator plasmid.

### Flow cytometry

To sort Siglec15^+^ cells, whole bone marrow was collected from mouse femurs and tibias after mice were euthanized with an overdose of inhaled isoflurane. Whole bone marrow cells were also used for culture and induction of BMMs using M-CSF (50 ng·mL^−1^) for higher Siglec15^+^ cell enrichment in further studies. After red blood cell lysis, the cell number was counted, and the same numbers of cells were incubated with a biotinylated mouse Siglec15-specific antibody (PA5-48221, Thermo Fisher Scientific). The primary antibody was biotinylated using a One-Step Antibody Biotinylation Kit (130-093-385, Miltenyi Biotec, MA) in accordance with the manufacturer’s instructions. For Siglec15^+^ cell separation, cells were then labeled with a 1:5 dilution of MACS^®^ anti-biotin microBeads (130-090-485, Miltenyi Biotec, MA) for 15 m. Each sample (8 × 10^6^ antibody- and microbead-labeled cells) was magnetically sorted at room temperature using “MS” columns inserted into a Miltenyi OctoMACS separator (130-042-201 and 130-042-109, Miltenyi Biotec). The cells were then placed on ice before counting. The Siglec15^+^ and Siglec15^−^ cell distributions were analyzed and quantified by flow cytometry. Cells were incubated with a conjugated anti-mouse Siglec15 antibody (PA5-48221, Thermo Fisher Scientific) for 30 min on ice. The conjugation of the primary antibody was performed using an Atto633 Conjugation Kit (ab269898, Abcam) in accordance with the manufacturer’s instructions. The cells were then sorted for Atto633 enrichment after live/dead cell sorting.

### Statistical analysis

All data presented in this study were generated from at least three repeated assays unless otherwise indicated. SPSS software (Ver. 20.0) and Prism 8.0 software (GraphPad) were used for statistical analysis. Differences were considered statistically significant at *P* < 0.05. Error bars in the plots represent the standard deviation (SD).

## Supplementary information

Supplementary materials

Uncropped gels
