# Supplementary material for: Sialylation of TLR2 initiates osteoclast fusion
Source: Bone Res. 2022 Mar 2;10:24. doi: 10.1038/s41413-022-00186-0 (PMC8888621; doi:10.1038/s41413-022-00186-0)
Supplement: Supplementary file 1 — Supplementary materials [file 41413_2022_186_MOESM1_ESM.docx]

Supplementary Materials for

**Sialylation of TLR2 Initiates Osteoclast Fusion**

Ce Dou^1,2^, Gehua Zhen^1^, Yang Dan^1^, Mei Wan^1^, Nathachit Limjunyawong^3^, Yutong Wu ^2^, Linda Liu^4^, Jianzhong Xu^2*^, Shiwu Dong^2*^, Xu Cao^1^*

^1^ Department of Orthopedic Surgery, Institute of Cell Engineering and Department of Biomedical Engineering, Johns Hopkins University School of Medicine, Baltimore, Maryland 21205, USA.

^2^ Department of Orthopedics, Southwest Hospital, Third Military Medical University, Chongqing 400038, China.

^3^ The Solomon H. Snyder Department of Neuroscience, Johns Hopkins University School of Medicine, Baltimore, Maryland 21205, USA.

^4^ NextCure Inc, Beltsville, MD, USA.

*Correspondence to: Xu Cao: xcao11@jhmi.edu; Shiwu Dong: dongshiwu@tmmu.edu.cn; Jianzhong Xu: xujianzhong1962@163.com

**This file includes:**

Figures S1-S13

Supplementary texts

Figure S14-S20

References (*58*)

**Supplementary Figures:**


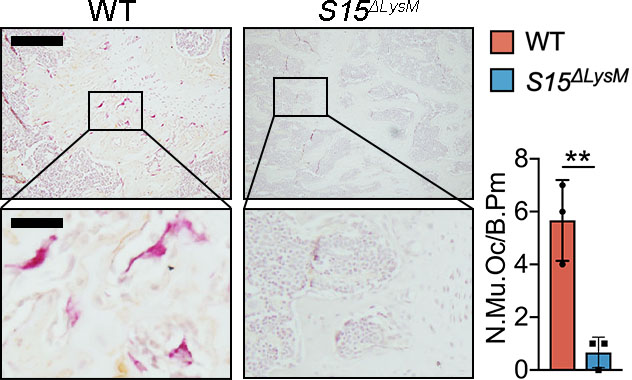


Supplementary Figure 1. TRAP stain of distal 11 wk-old *Siglec15*^fl/fl^ (WT) and *Siglec15*^ΔLysM^ mouse femur sections and quantification of multinuclear osteoclast number, n = 3. Bar represents 200 μm. Data represents Mean ± SD, statistically significant differences are indicated as ** (*p* < 0.01).


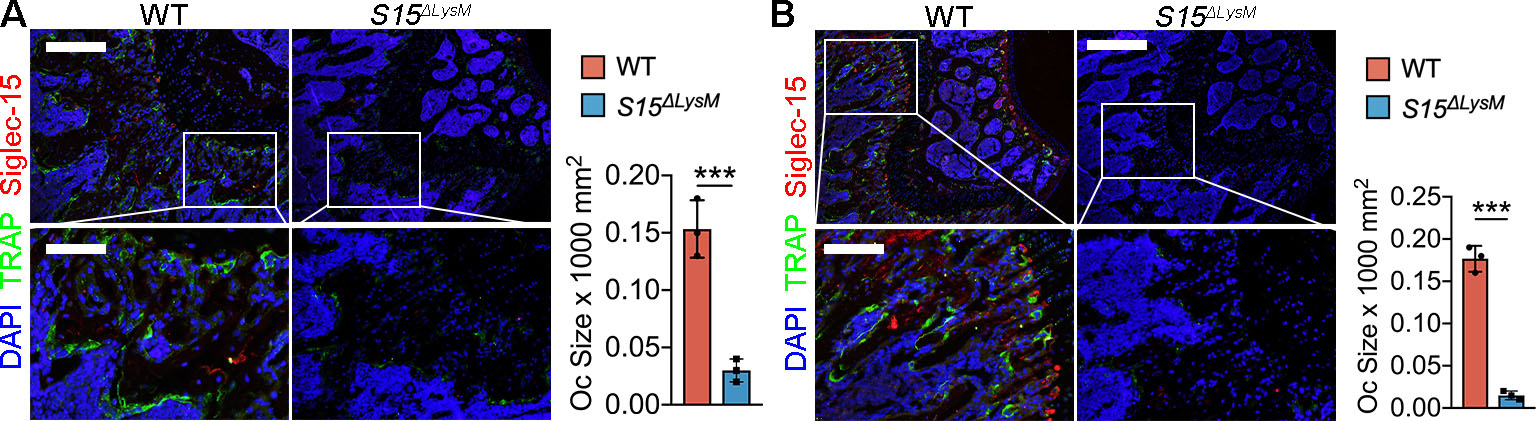


Supplementary Figure 2. Immunofluoresent stain of Siglec15 and TRAP of distal 4 wk-old (**A**) and 11 wk-old (**B**) *Siglec15*^fl/fl^ (WT) and *Siglec15*^ΔLysM^ mouse femur sections and quantification of osteoclast size, n = 3. Bar represents 200 μm. Data represents Mean ± SD, statistically significant differences are indicated as *** (*p* < 0.001).


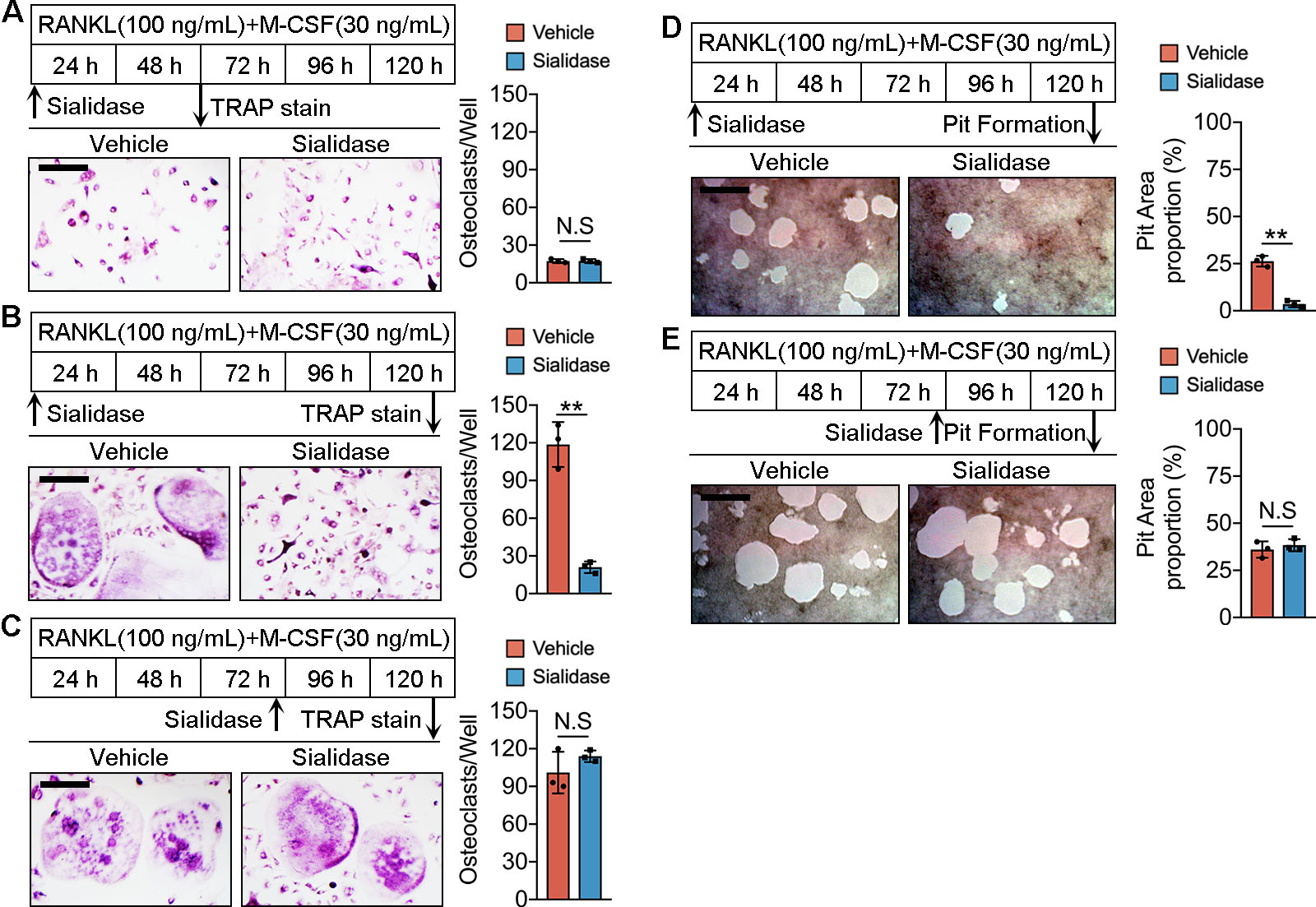


Supplementary Figure 3. WT BMMs were treated with vehicle or sialidase. Sialidase was treated at the beginning and TRAP stain was performed at 48 h (**A**) and 120 h (**B**). Sialidase was treated at 72 h and TRAP stain was performed at 120 h (**C**). Quantification of osteoclast number per well on the right. n = 3. Sialidase was treated at the beginning and pit formation was detected at 120 h (**D**), sialidase was treated at 72 h and pit formation was detected at 120 h (**E**). n = 3. Bar represents 100 μm. Data represents Mean ± SD, statistically significant differences are indicated as *** (*p* < 0.001).


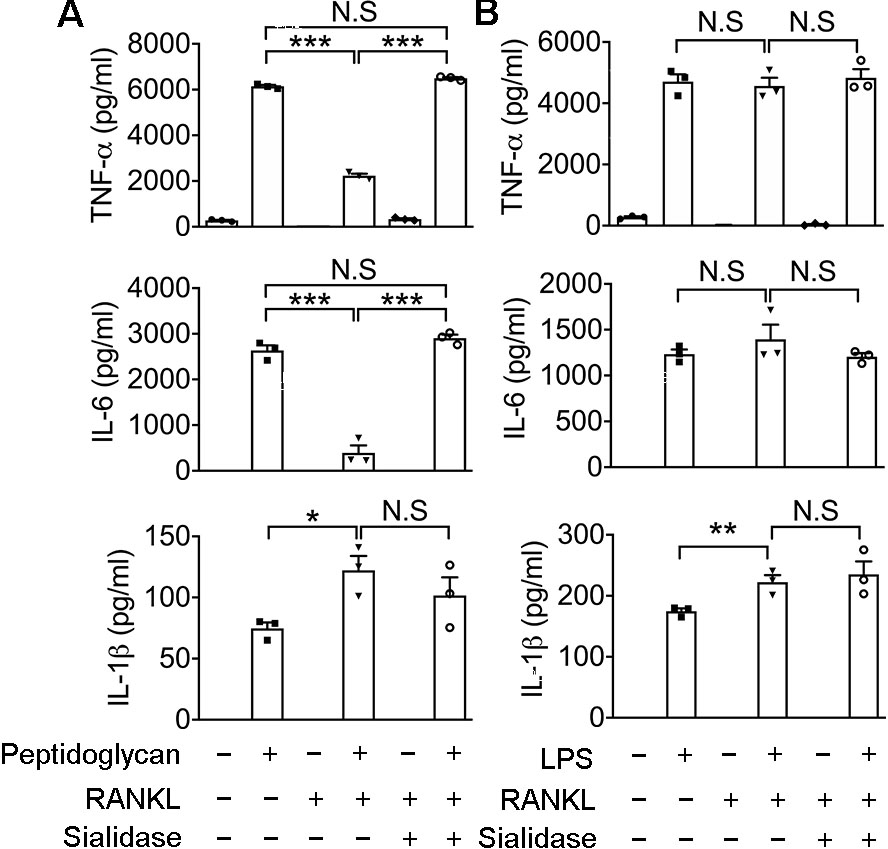


Supplementary Figure 4. ELISA detection of TNF-alpha, IL-6 and IL-1beta in WT BMMs treated with peptidoglycan (A) or LPS (B) as indicated. n = 3. Data represents Mean ± SD, statistically significant differences are indicated as *** (*p* < 0.001).


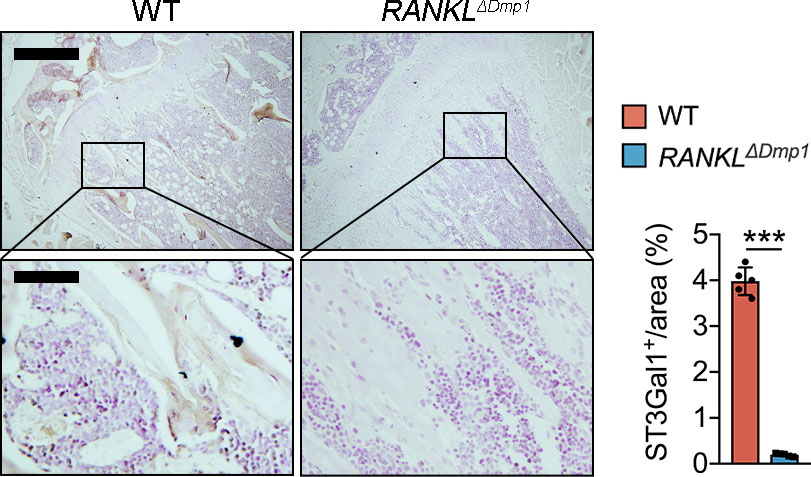


Supplementary Figure 5. Immunohistochemistry (IHC) staining of ST3Gal1 in *RANKL^fl/fl^* and *RANKL*^ΔDmp^ mice distal femurs. Bar represents 200 μm. Quantification of ST3Gal1 positive area is shown on the right. n = 3. Data represents Mean ± SD, statistically significant differences are indicated as * (*p* < 0.05), *** (*p* < 0.001).


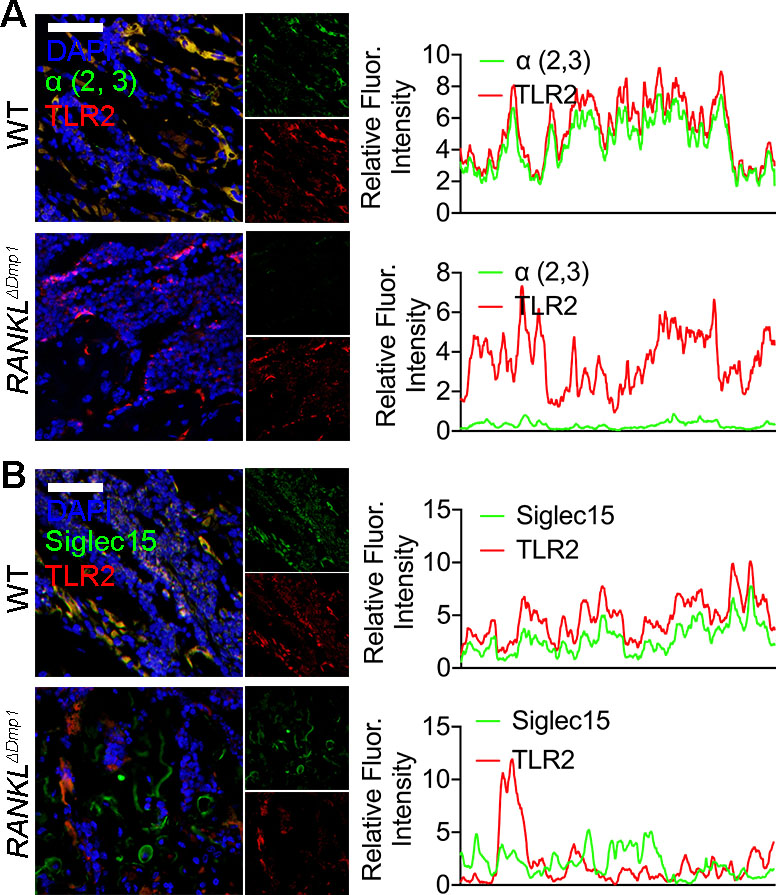


Supplementary Figure 6. (**A**) Immunostaining of α(2,3) and TLR2 of distal femur sections of *RANKL^fl/fl^* and *RANKL*^ΔDmp^ mice and co-localization analysis of α(2,3) with TLR2. (**B**) Immunostaining of Siglec15 and TLR2 of distal femur sections of *RANKL^fl/fl^* and *RANKL*^ΔDmp^ mice and co-localization analysis of Siglec15 with TLR2.


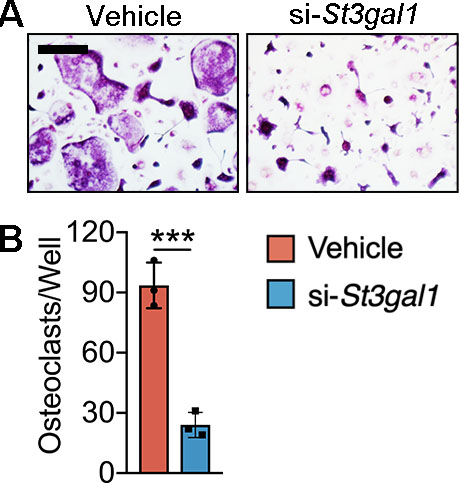


Supplementary Figure 7. (**A**) TRAP staining of RAW264.7 cells transfected with *St3gal1* siRNA or vehicle before RANKL stimulation for 72 h. (**B**) Quantification of osteoclast number per well, n = 3. Data represents Mean ± SD, statistically significant differences are indicated as *** (*p* < 0.001).


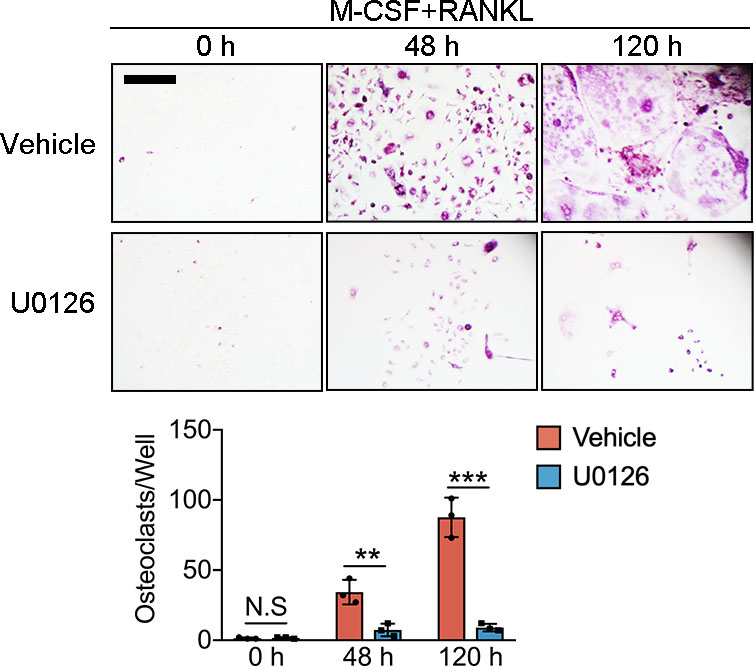


Supplementary Figure 8. TRAP stain of WT BMMs treated with vehicle or U0126 during osteoclastogenesis. Bar represents 100 μm. Quantification of osteoclast number per well. n = 3. Data represents Mean ± SD, statistically significant differences are indicated as *** (*p* < 0.001).


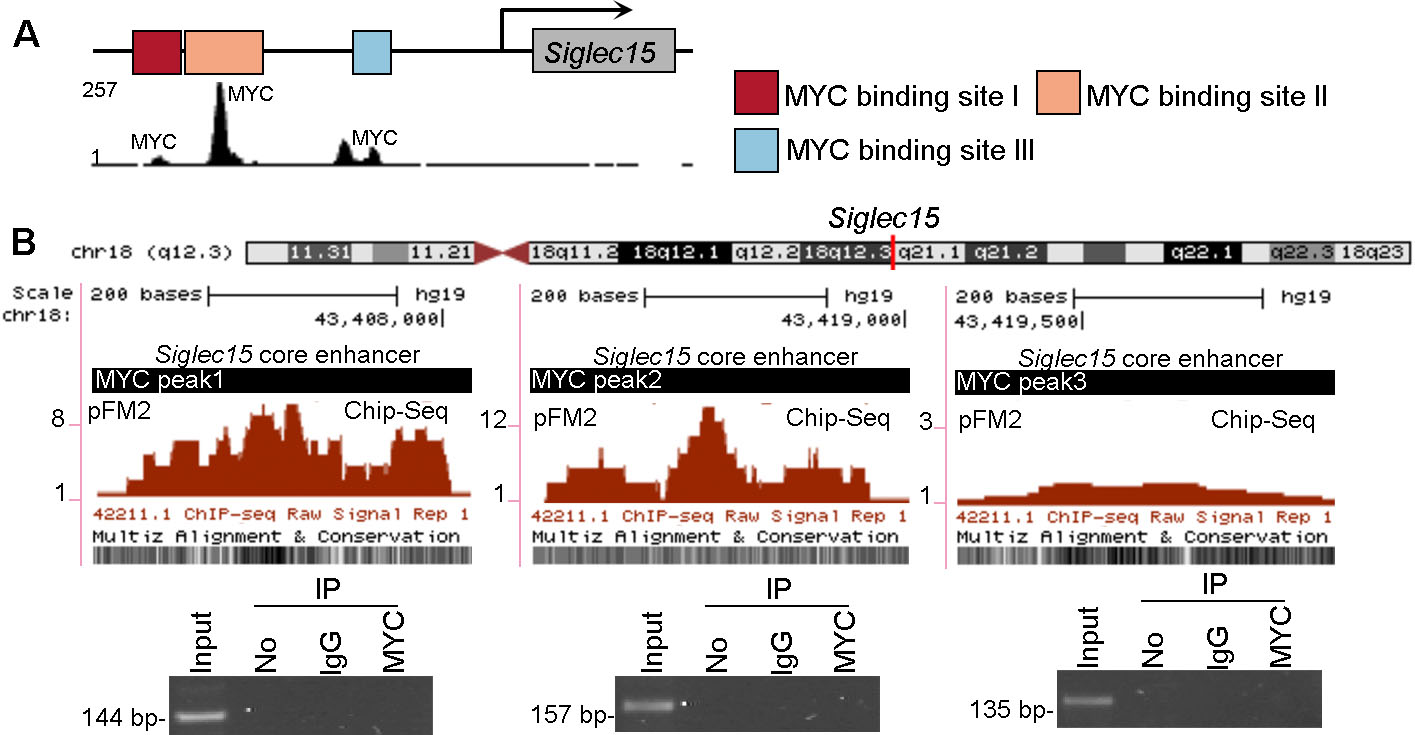


Supplementary Figure 9. (A) Three MYC recognition sites at the *Siglec15* core enhancer. (B) chromatin immunoprecipitation (ChIP) assay validation of the direct MYC binding induced by GM-CSF.


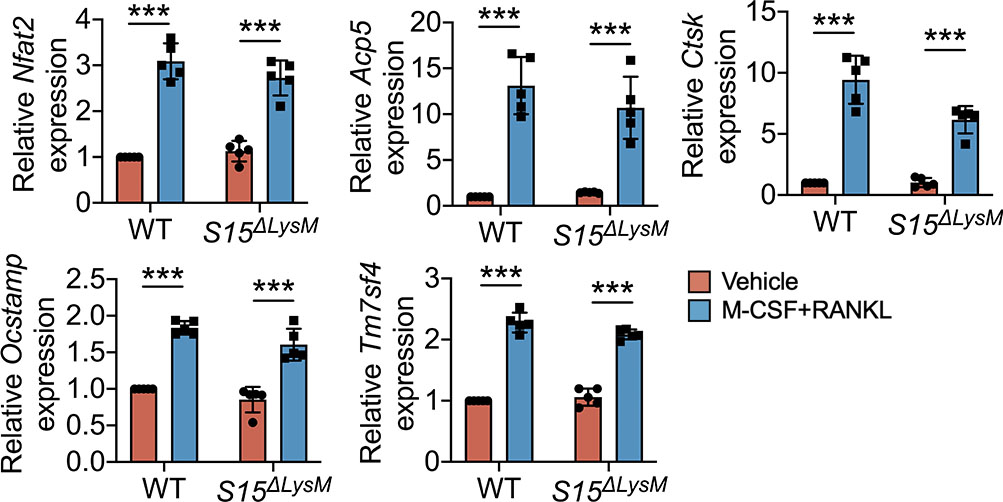


Supplementary Figure 10. Relative expression of *Nfat2*, *Acp5*, *ctsk*, *oc-stamp* and *Tm7sf4* in WT and *Siglec15*^ΔLysM^ bone marrow macrophages treated with or without M-CSF+RANKL, n = 5. Data represents Mean ± SD, statistically significant differences are indicated as *** (*p* < 0.001).


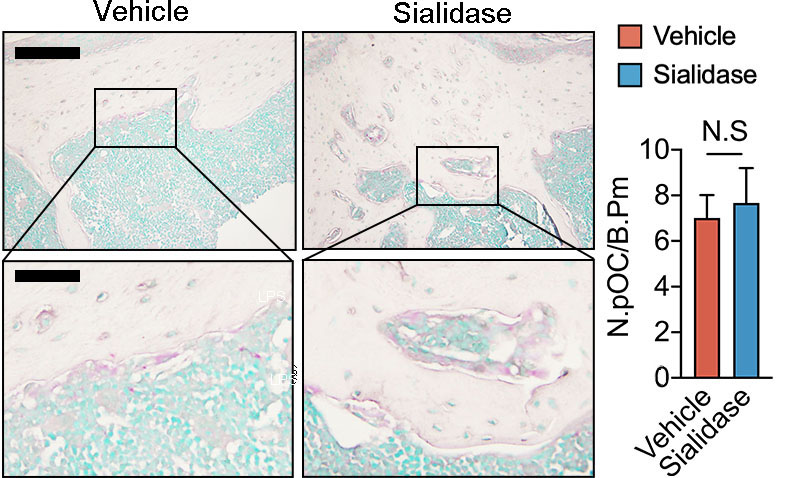


Supplementary Figure 11. TRAP staining of distal femur sections of *Siglec15*^ΔLysM^ mice intrafemorally injected with vehicle or sialidase and quantification of multinuclear osteoclast number, n = 3.


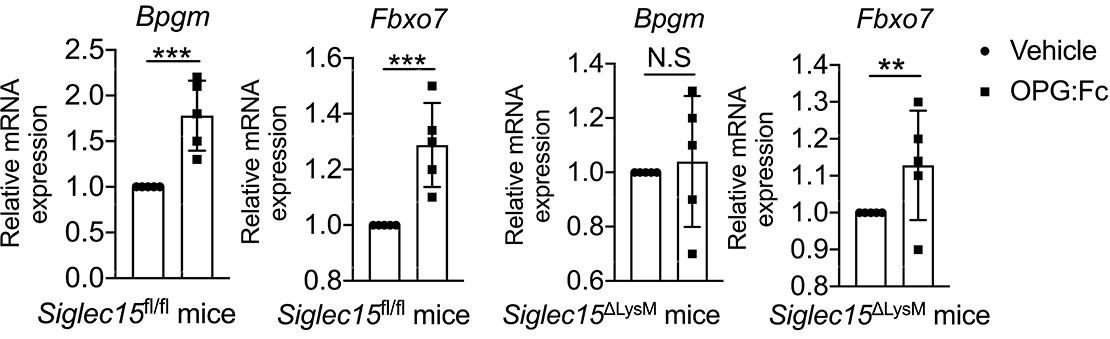


Supplementary Figure 12. Relative expression of osteomorph marker genes Bpgm and Fbxo7 in *Siglec15*^fl/fl^ and *Siglec15*^ΔLysM^ mice with or without OPG:Fc treatments, n = 5. Data represents Mean ± SD, statistically significant differences are indicated as ** (*p* < 0.01), *** (*p* < 0.001).


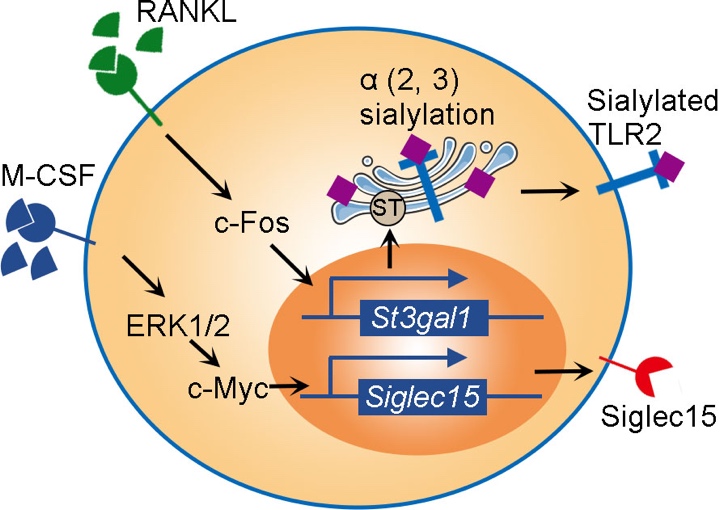


Supplementary Figure 13. Schematic diagram showing that Siglec15 is activated by M-CSF via ERK1/2-cMyc signaling and St3gal1 is activated by RANKL via c-Fos dependent pathway.

**Supplementary Texts**

To understand the detailed mechanism of Siglec15 regulating osteoclast formation, we incubated WT BMMs with biotinylated Siglec15 monoclonal antibody and manually separated Siglec15^+^ BMMs from Siglec15^−^ BMMs using anti-biotin microbeads and magnetic separators (Fig. S11A). Separated Siglec15^+^ and Siglec15^−^ BMMs were then stimulated with M-CSF and RANKL for osteoclastogenesis. Surprisingly, no significant difference was observed in osteoclast formation capacity between Siglec15^+^ and Siglec15^−^ BMMs (Fig. S12). One study reported that osteoclast fusion might be initiated by a small subset of progenitors ^58^; therefore, we combined separated Siglec15^+^ BMMs with BMMs isolated from *Siglec15*^ΔLysM^ mice in different proportions (1%, 5%, and 10%). TRAP staining results showed that Siglec15^+^ BMMs failed to initiate osteoclast fusion in BMMs deficient of Siglec15 (Fig. S13). Furthermore, transwell culture showed that Siglec15^+^ BMMs also have no indirect effects on osteoclastogenesis of WT, Siglec15^−^, and Siglec15^−/−^ BMMs (Fig. S14). Immunofluorescent staining confirmed that Siglec15^+^ and Siglec15^−^ BMMs can form multinucleated osteoclasts with normal actin rings, and the expression of Siglec15 was restored in Siglec15^−^ BMMs during osteoclastogenesis (Fig. S15). Separated Siglec15^−^ BMMs were then cultured with M-CSF, and FCM analysis showed that Siglec15^−^ BMMs restored Siglec15 expression after 3 days of stimulation (Fig. S11B, C). BMMs cultured with RANKL alone, absent M-CSF, cannot survive during osteoclastogenesis (Fig. S11D, E). Immunofluorescent staining results showed that M-CSF stimulation significantly increased the proportion of Siglec15^+^ BMMs in *Siglec15*^fl/fl^ mice (Fig. S11F, G). We then tested the other CSF family member, granulocyte-macrophage CSF (GM-CSF, encoded by *CSF2*), and found that GM-CSF cannot induce osteoclastogenesis (Fig. S16) nor the expression of Siglec15 (Fig. S17).


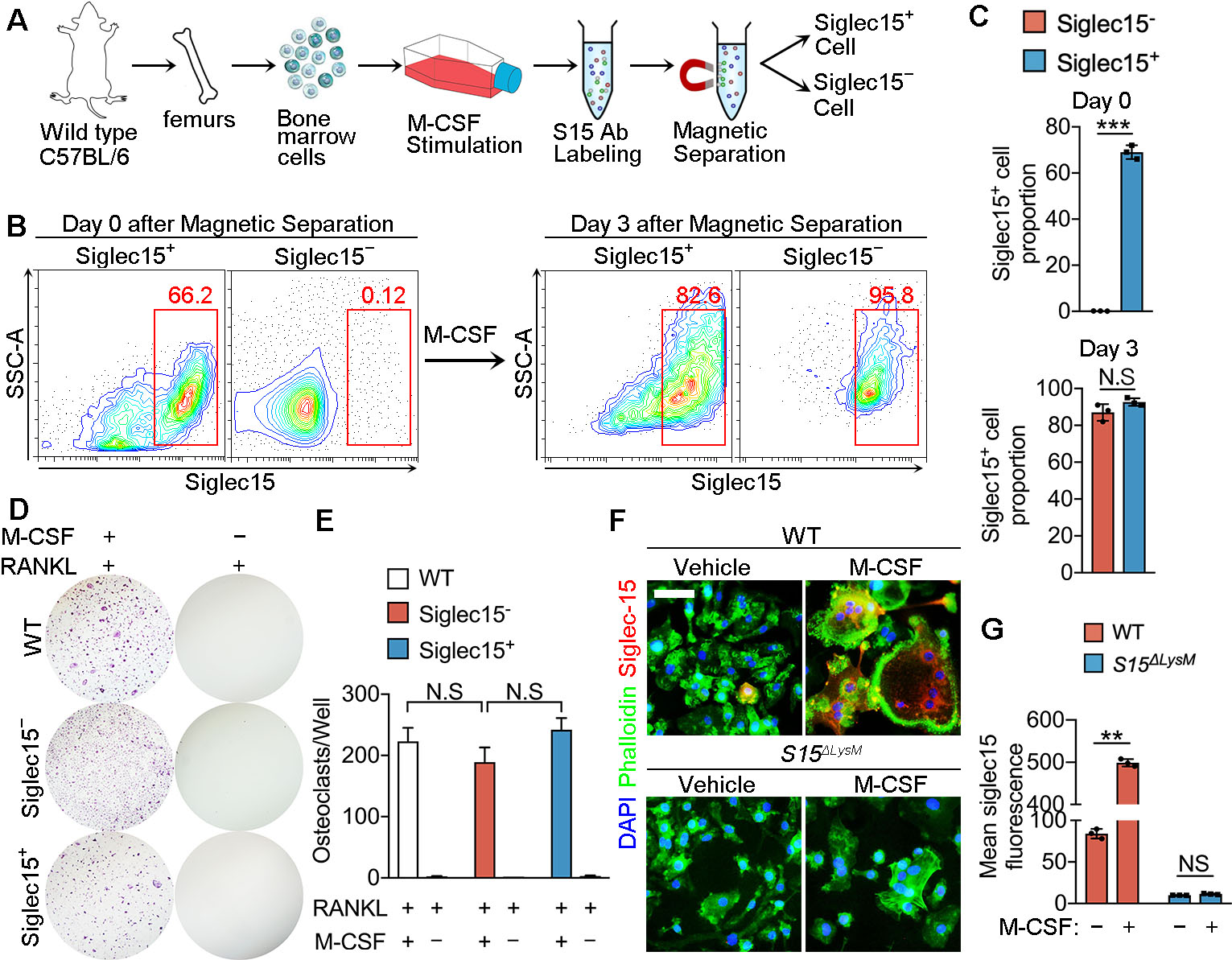


Supplementary Figure 14**. Siglec15 expression is stimulated by M-CSF.** (**A**) BMMs incubated with biotinylated Siglec15 monoclonal antibody were manually separated to Siglec15^+^ BMMs and Siglec15^−^ BMMs using anti-biotin microbeads and magnetic separators. (**B**) Flow cytometry analysis of WT and separated Siglec15− BMMs on day 0 and day 3 after treatment of M-CSF. (**C**) Quantification of Siglec15+ cell proportion. (**D**) TRAP stain of WT, Siglec15^−^ and Siglec15^+^ BMMs treated with M-CSF+RANKL or RANKL alone. (**E**) Site-directed mutagenesis of the MYC binding sites at *Siglec15* core enhancer. (**F**) Quantification of osteoclast number per well. (**G**) Quantification of mean Siglec15 fluorescent intensity was shown on the right. n = 3. Data represents Mean ± SD, statistically significant differences are indicated as ** (*p* < 0.01), *** (*p* < 0.001).


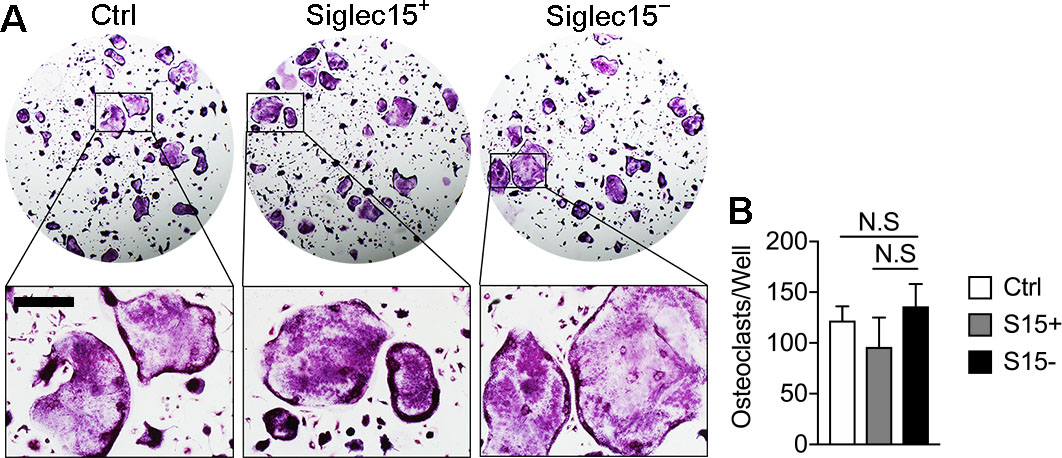


Supplementary Figure 15. (**A**) TRAP staining of Siglec15^+^ and Siglec15^−^ BMMs induced by RANKL and M-CSF. Bar represents 50 μm. (B) Quantification of osteoclast number per well, n = 3. Data represents Mean ± SD.


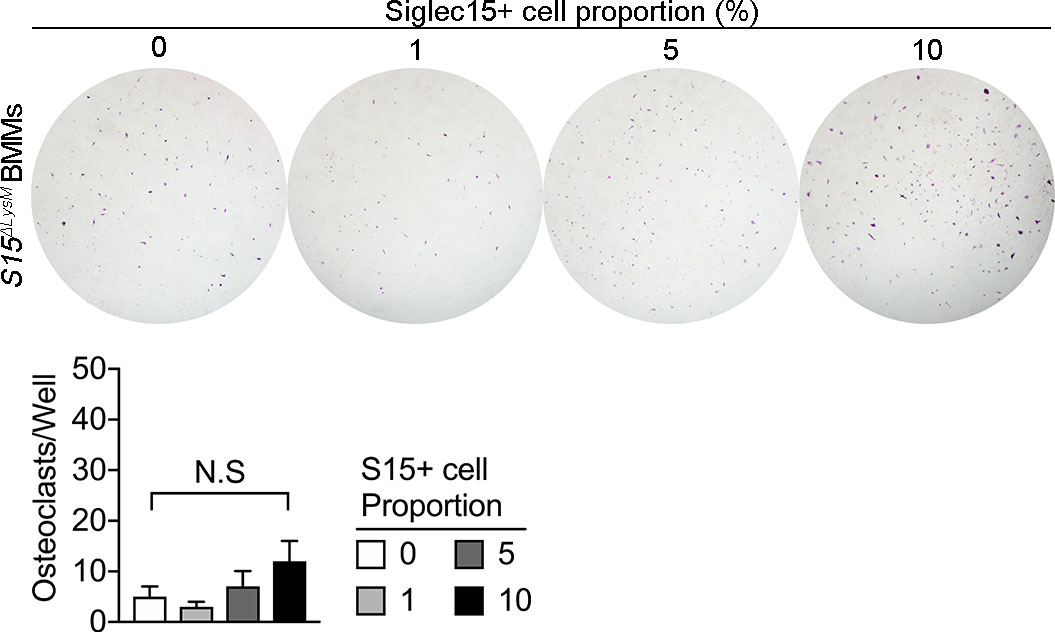


Supplementary Figure 16. TRAP stain of *Siglec15*^ΔLysM^ BMMs introduced with Siglec15^+^ BMMs of different proportion (0, 1%, 5%, 10%). Quantification of osteoclast number per well is shown below. n = 3. Data represents Mean ± SD.


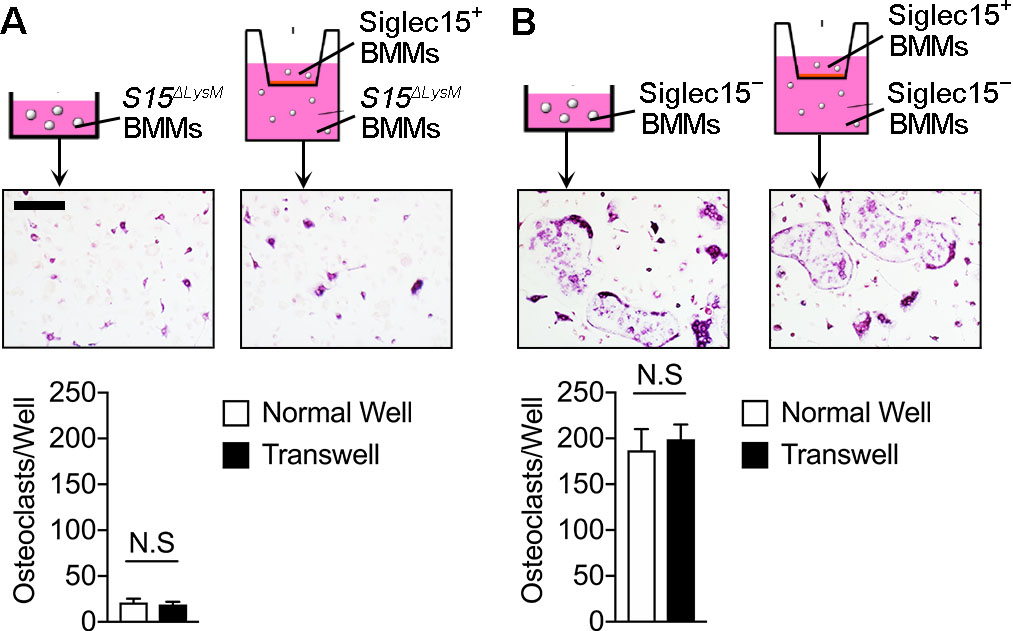


Supplementary Figure 17. (**A**) TRAP stain of *Siglec15*^ΔLysM^ BMMs in control wells or transwells co-incubated with Siglec15^+^ BMMs. Quantification of osteoclast number per well is shown below. Bar represents 50 μm. n = 3. Data represents Mean ± SD. (**B**) TRAP stain of Siglec15^−^ BMMs in control wells or transwells co-incubated with Siglec15^+^ BMMs. Quantification of osteoclast number per well is shown below. Bar represents 50 μm. n = 3. Data represents Mean ± SD.


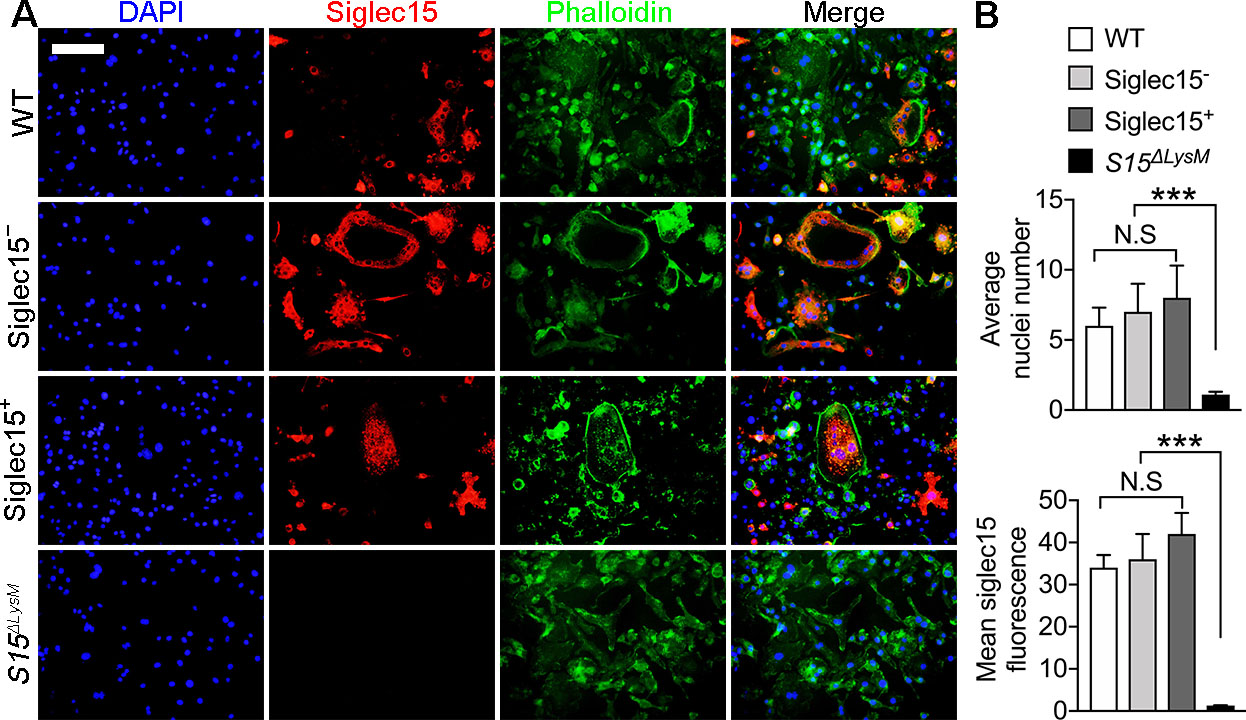


Supplementary Figure 18. (**A**) Immunostaining of Siglec15 and phalloidin in WT, Siglec15^−^, Siglec15^+^, and *Siglec15*^ΔLysM^ BMMs during osteoclastogenesis. Bar represents 50 μm. (**B**) Quantification of average nuclei number and mean siglec15 fluorescent intensity. Data represents Mean ± SD, statistically significant differences are indicated as *** (*p* < 0.001).


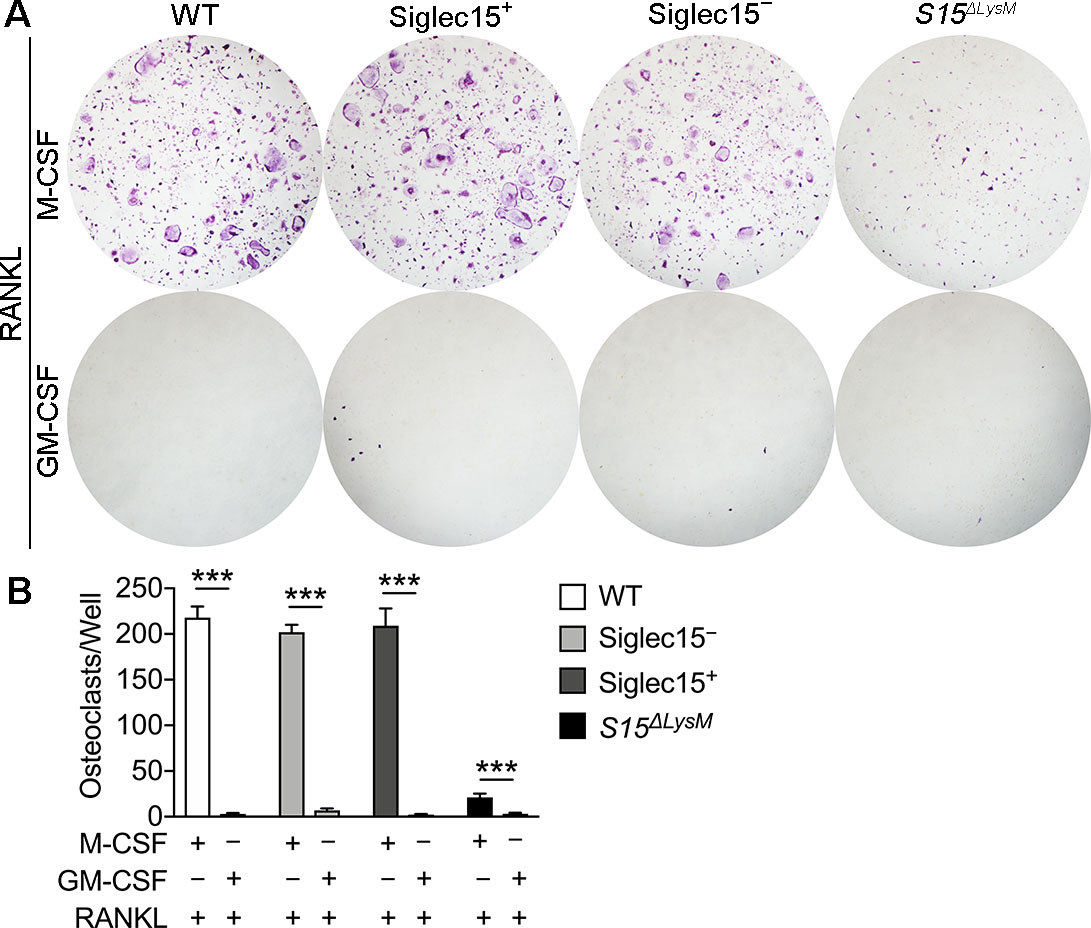


Supplementary Figure 19. (A) TRAP stain of WT, Siglec15^−^, Siglec15^+^, and *Siglec15*^ΔLysM^ BMMs induced with RANKL+M-CSF or RANKL+GM-CSF. (B) Quantification of osteoclast number per well on the right. n = 3. Data represents Mean ± SD, statistically significant differences are indicated as *** (*p* < 0.001).


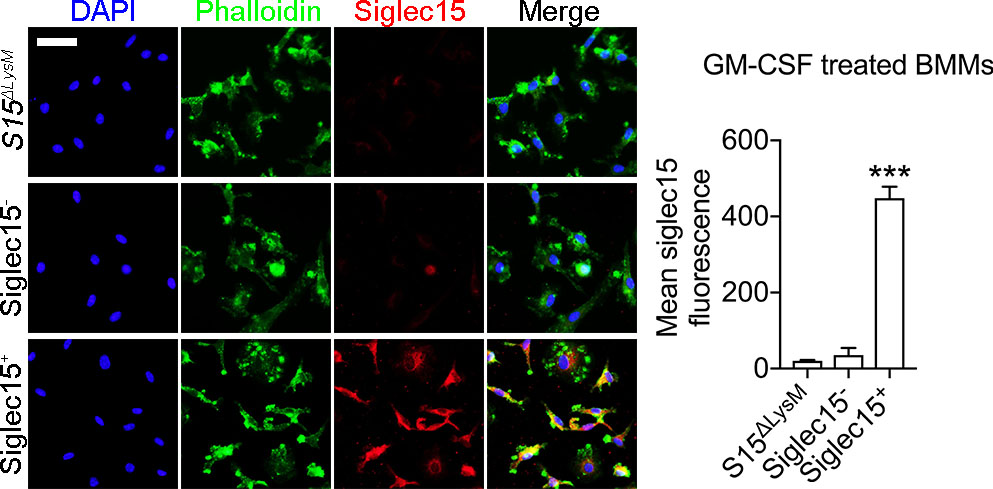


Supplementary Figure 20. Immunostaining of phalloidin and Siglec15 in Siglec15^−^, Siglec15^+^, and *Siglec15*^ΔLysM^ BMMs treated with GM-CSF. Quantification of mean Siglec15 fluorescence is shown on the right. Bar represents 50 μm. n = 3. Data represents Mean ± SD, statistically significant differences are indicated as *** (*p* < 0.001).

**References**

58. N. Levaot *et al.*, Osteoclast fusion is initiated by a small subset of RANKL-stimulated monocyte progenitors, which can fuse to RANKL-unstimulated progenitors. *Bone* **79**, 21-28 (2015).
