# Supplementary material for: Sialylation of TLR2 initiates osteoclast fusion
Source: Bone Res. 2022 Mar 2;10:24. doi: 10.1038/s41413-022-00186-0 (PMC8888621; doi:10.1038/s41413-022-00186-0)

Full unedited gel for Figure 1C

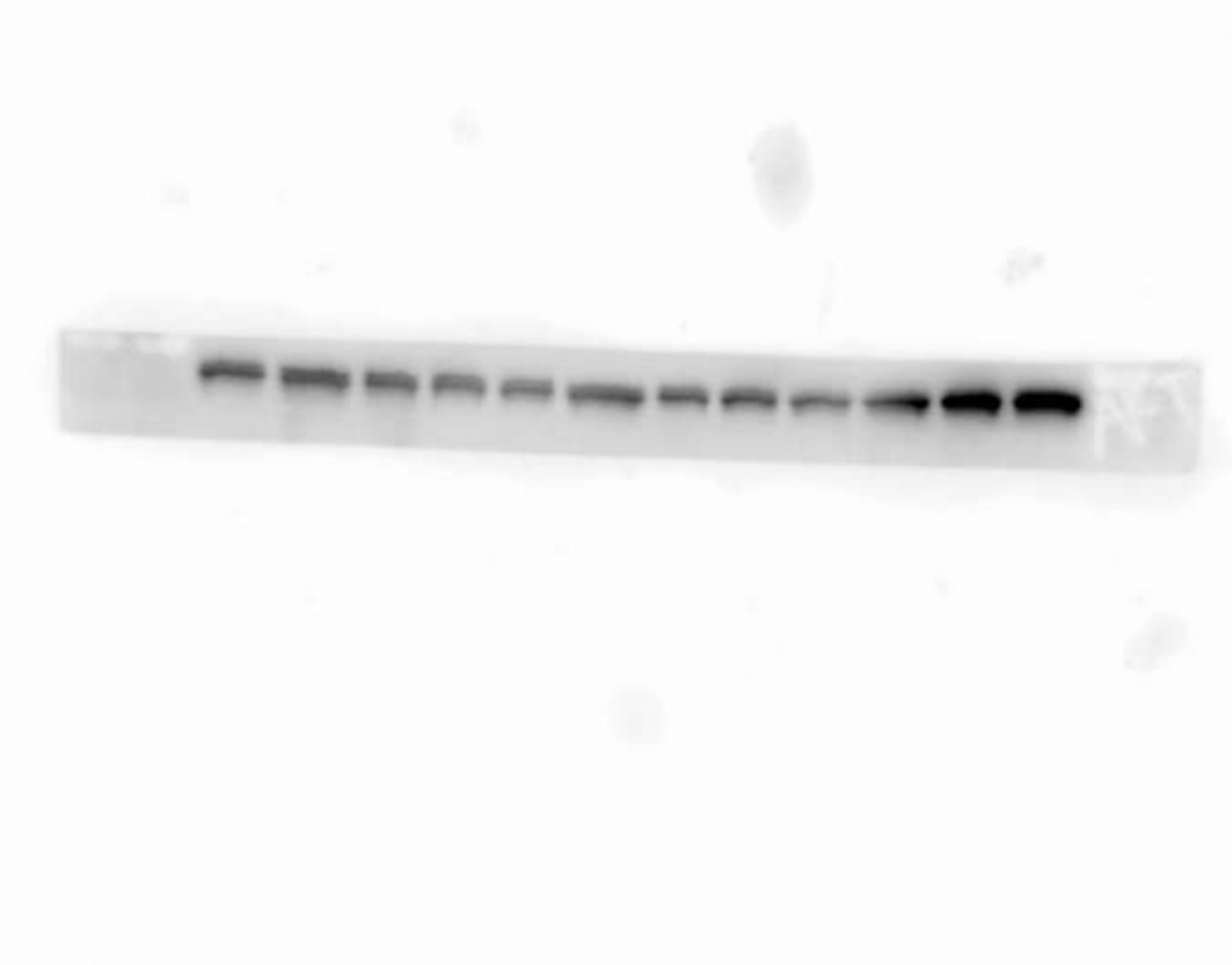

Full unedited gel for Figure 1C

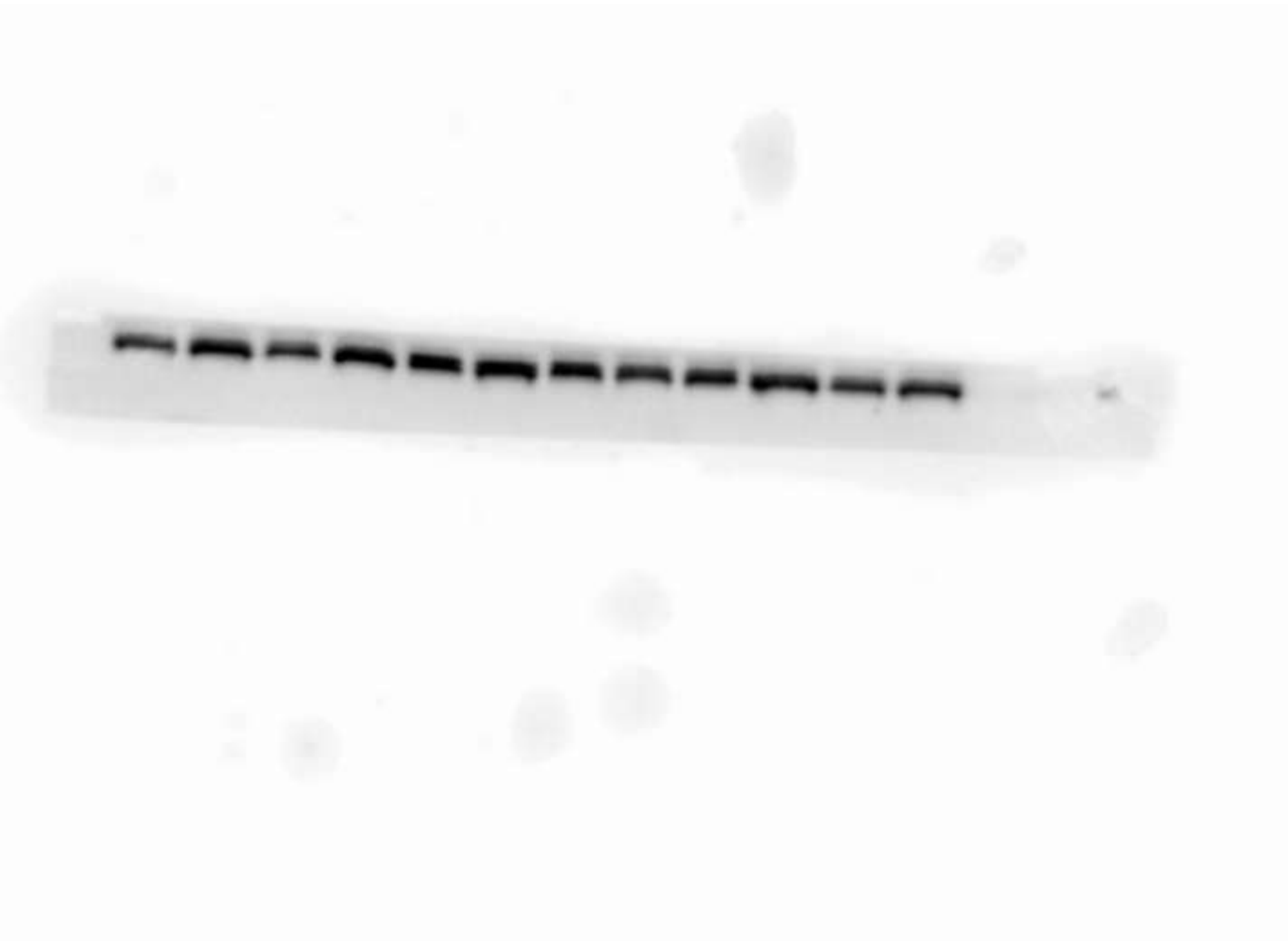

Full unedited gel for Figure 2D

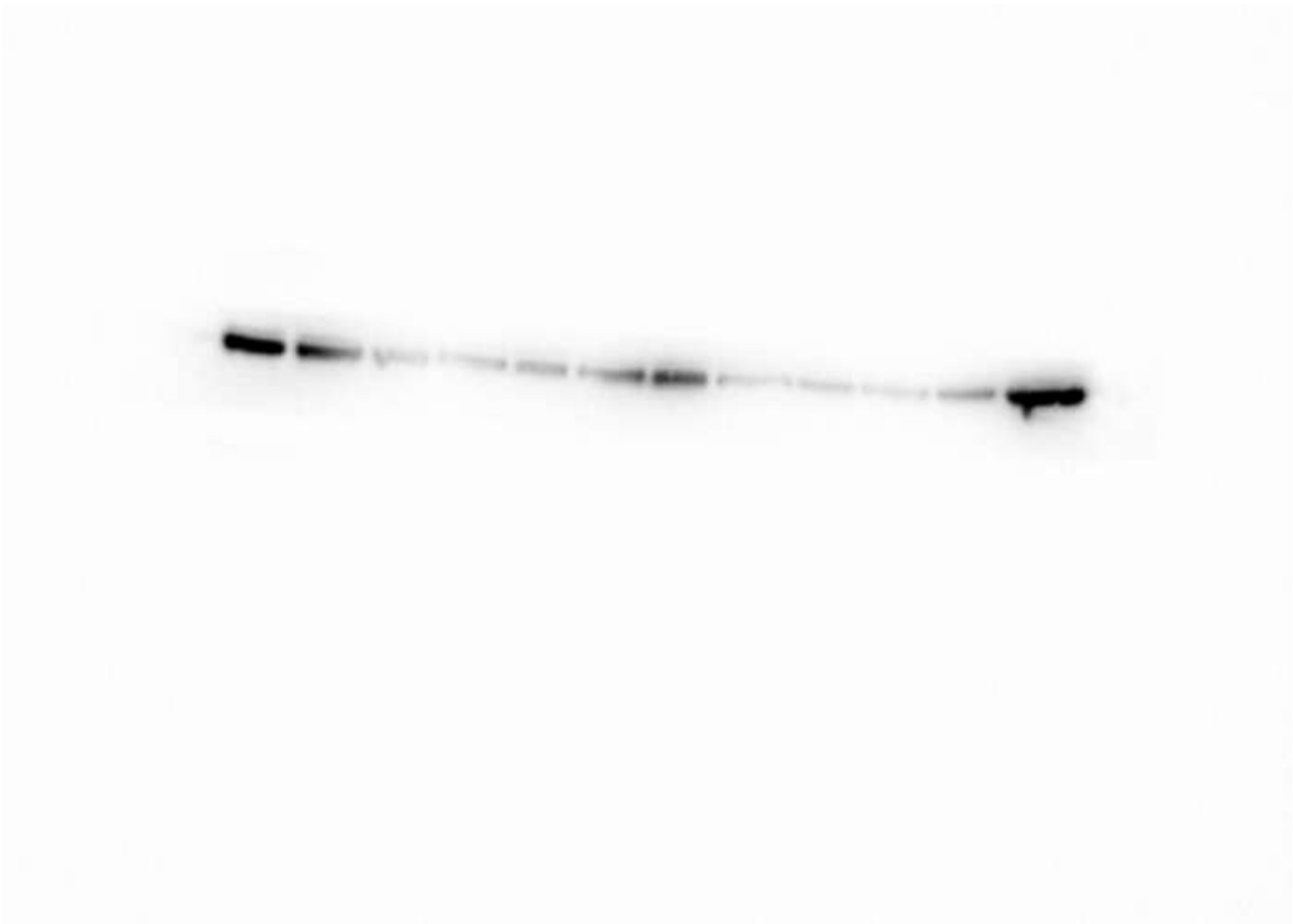

Full unedited gel for Figure 6H

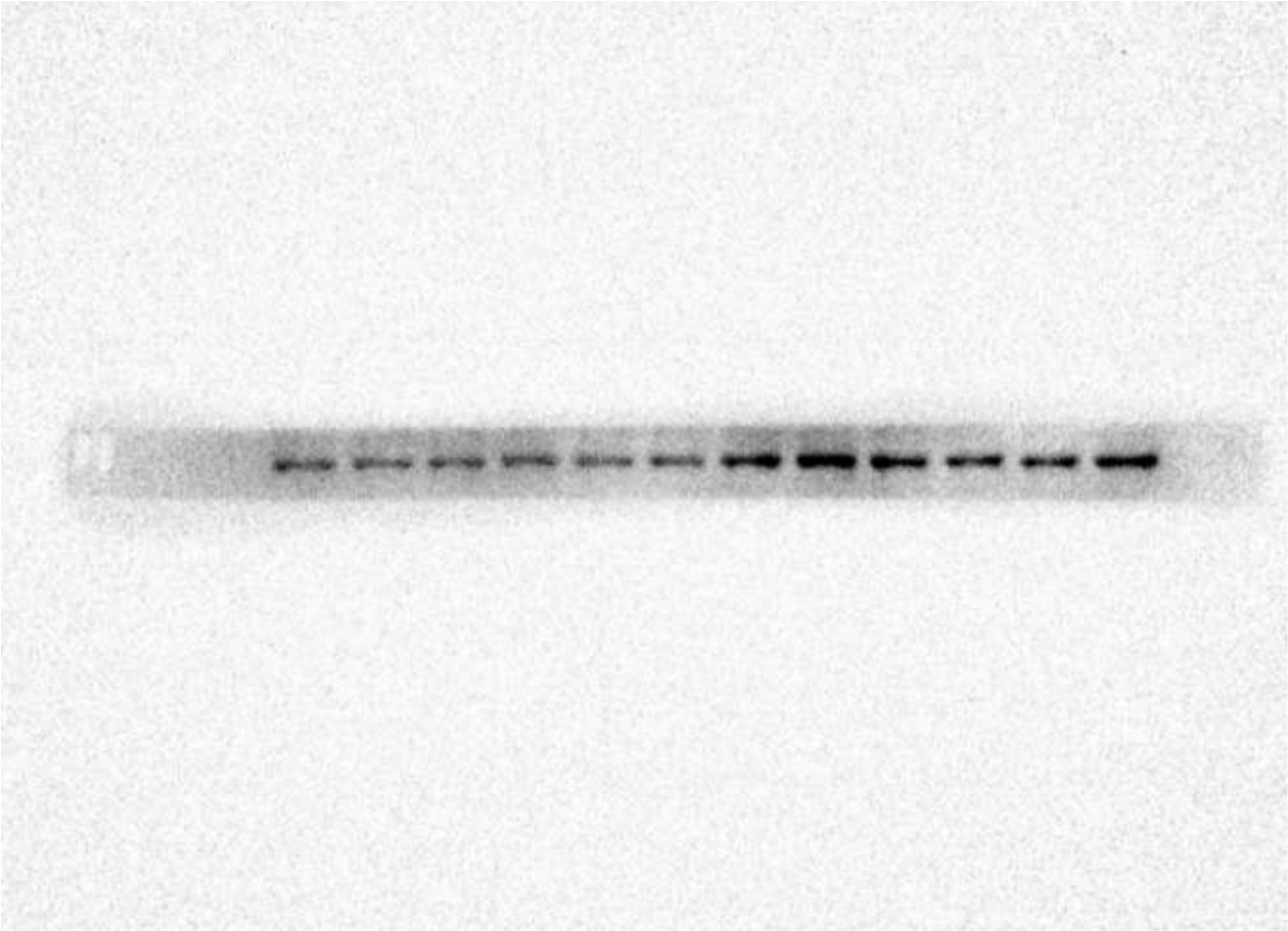

Full unedited gel for Figure 6E

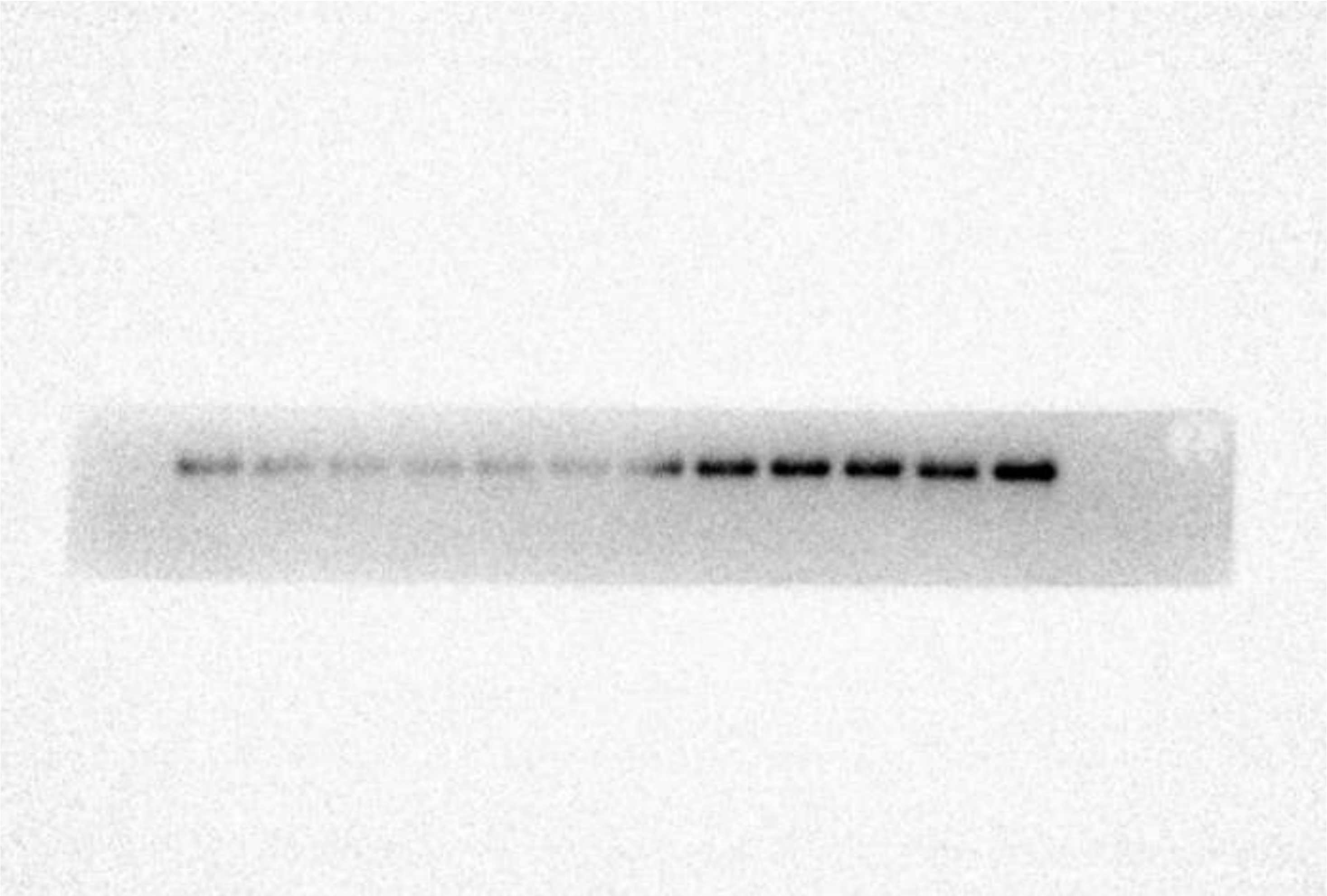

Full unedited gel for Figure 3C

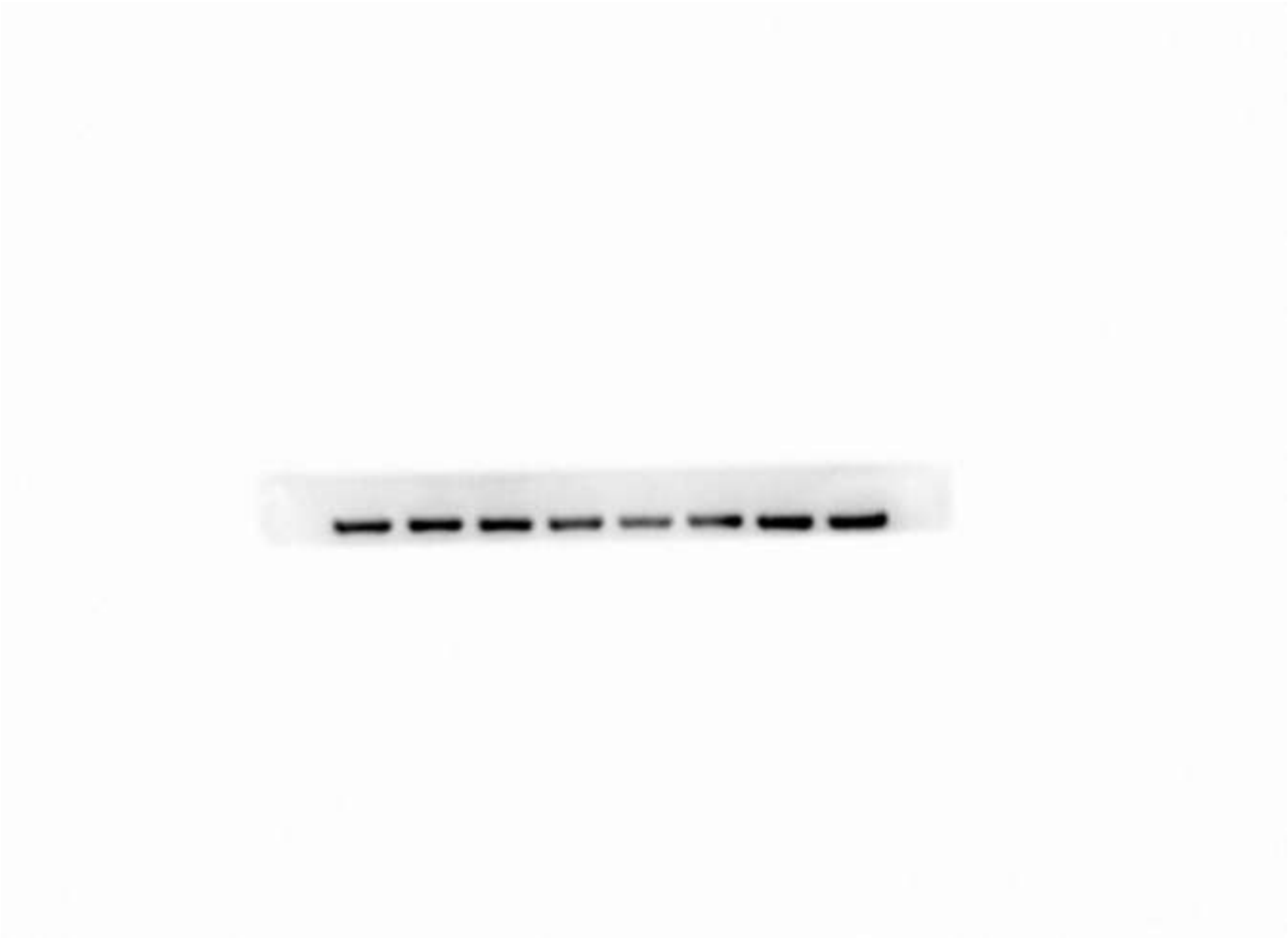

Full unedited gel for Figure 3C

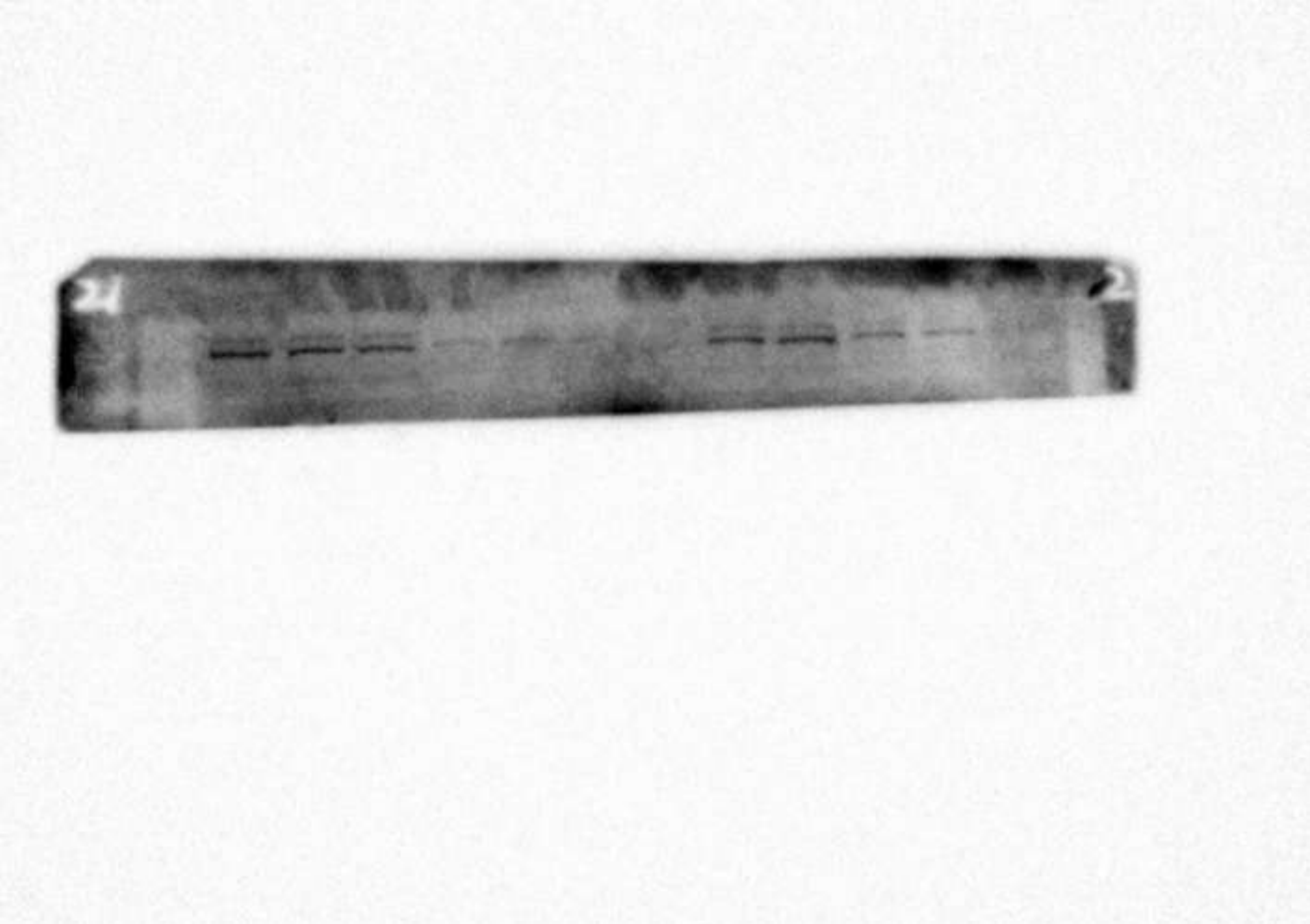

Full unedited gel for Figure 6C

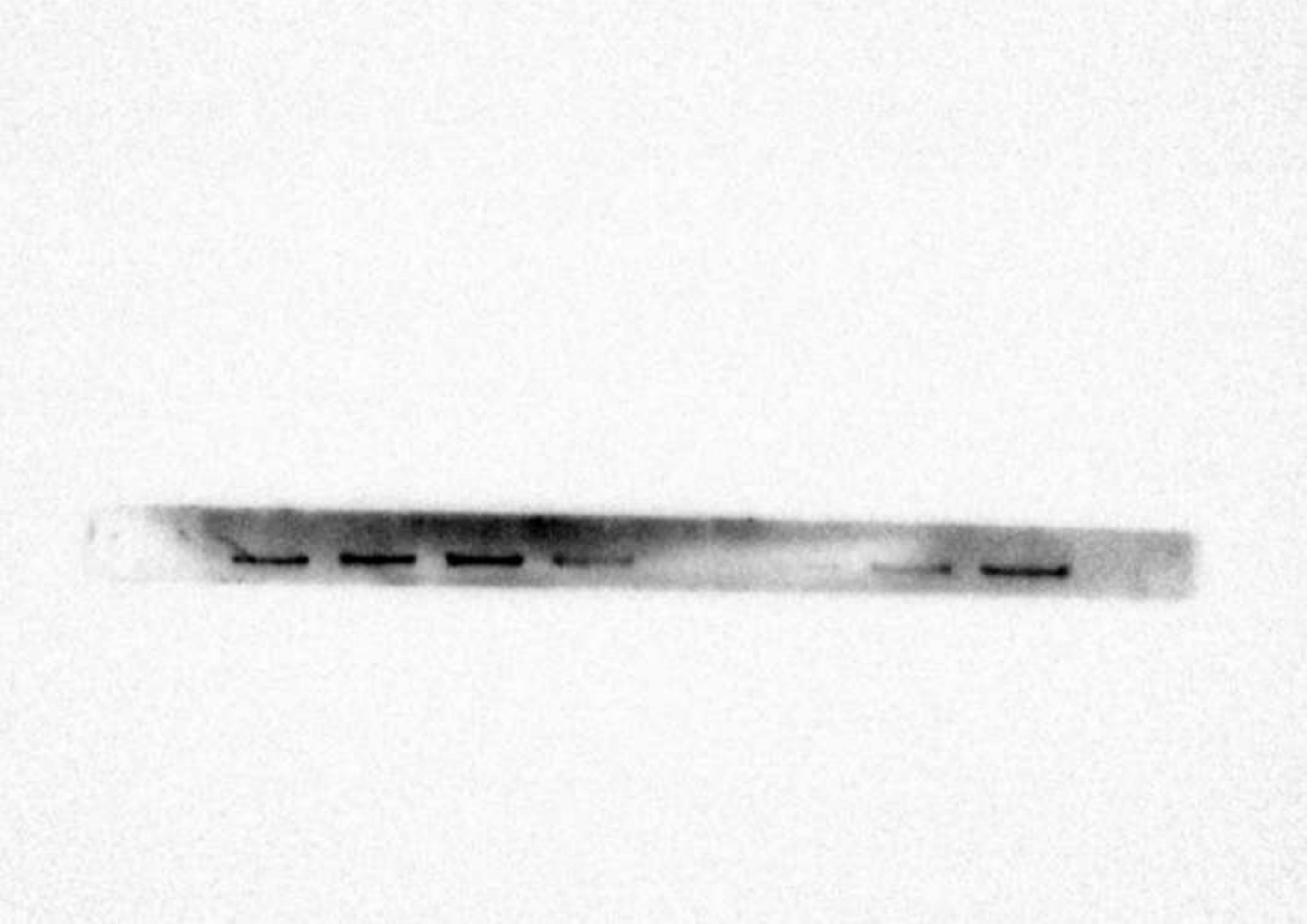

Full unedited gel for Figure 6E

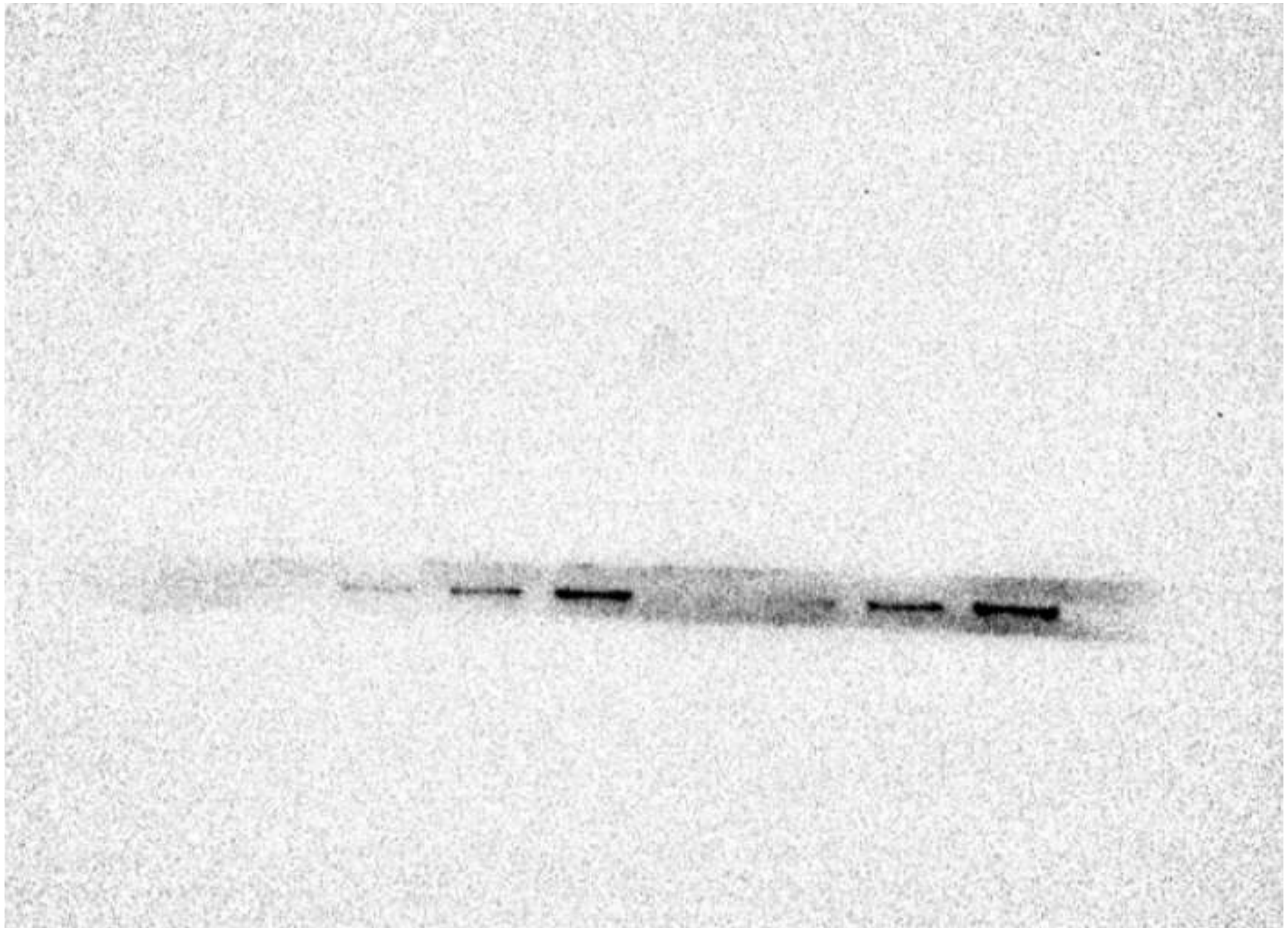

Full unedited gel for Figure 6H

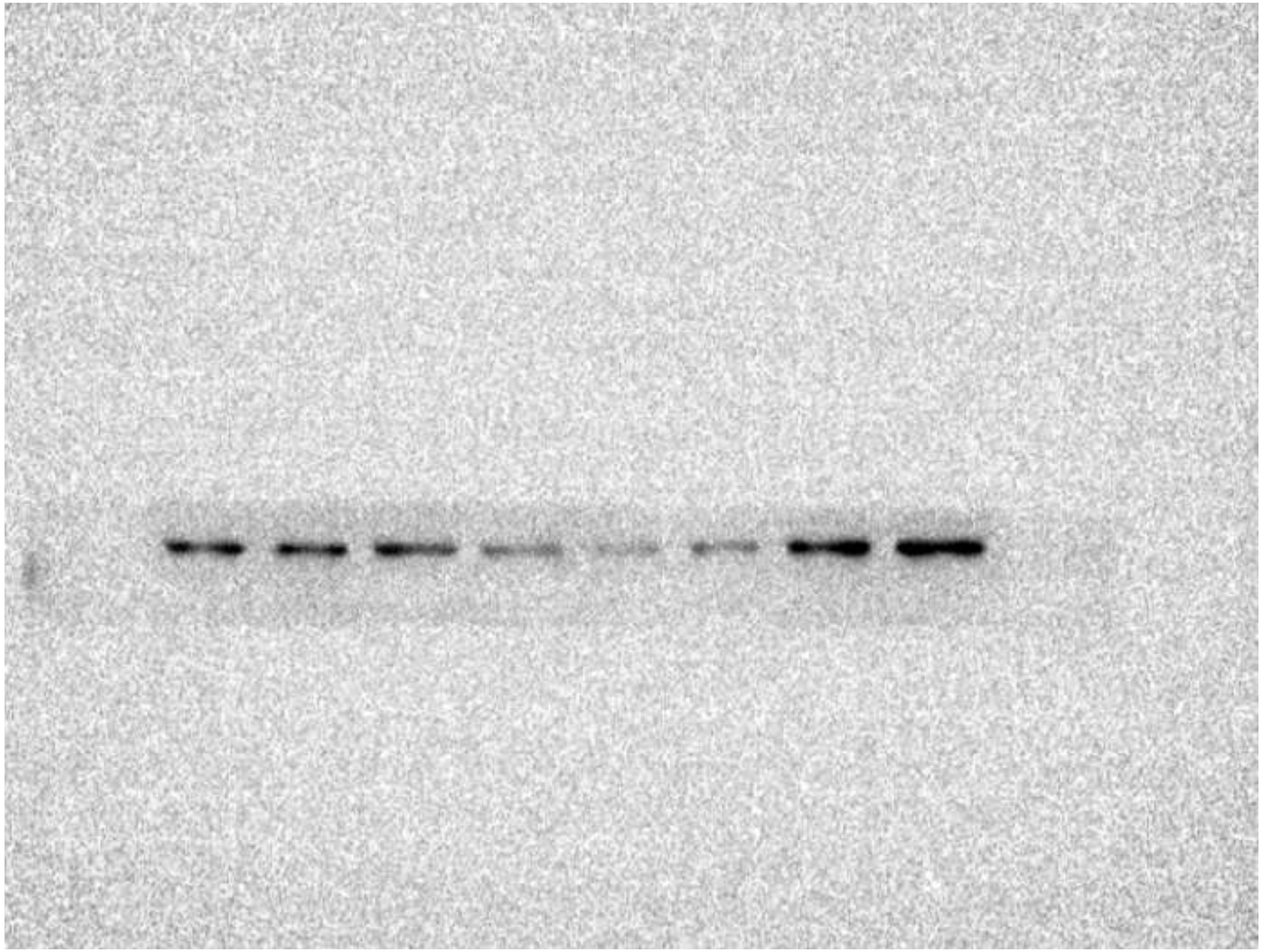

Full unedited gel for Figure 6C

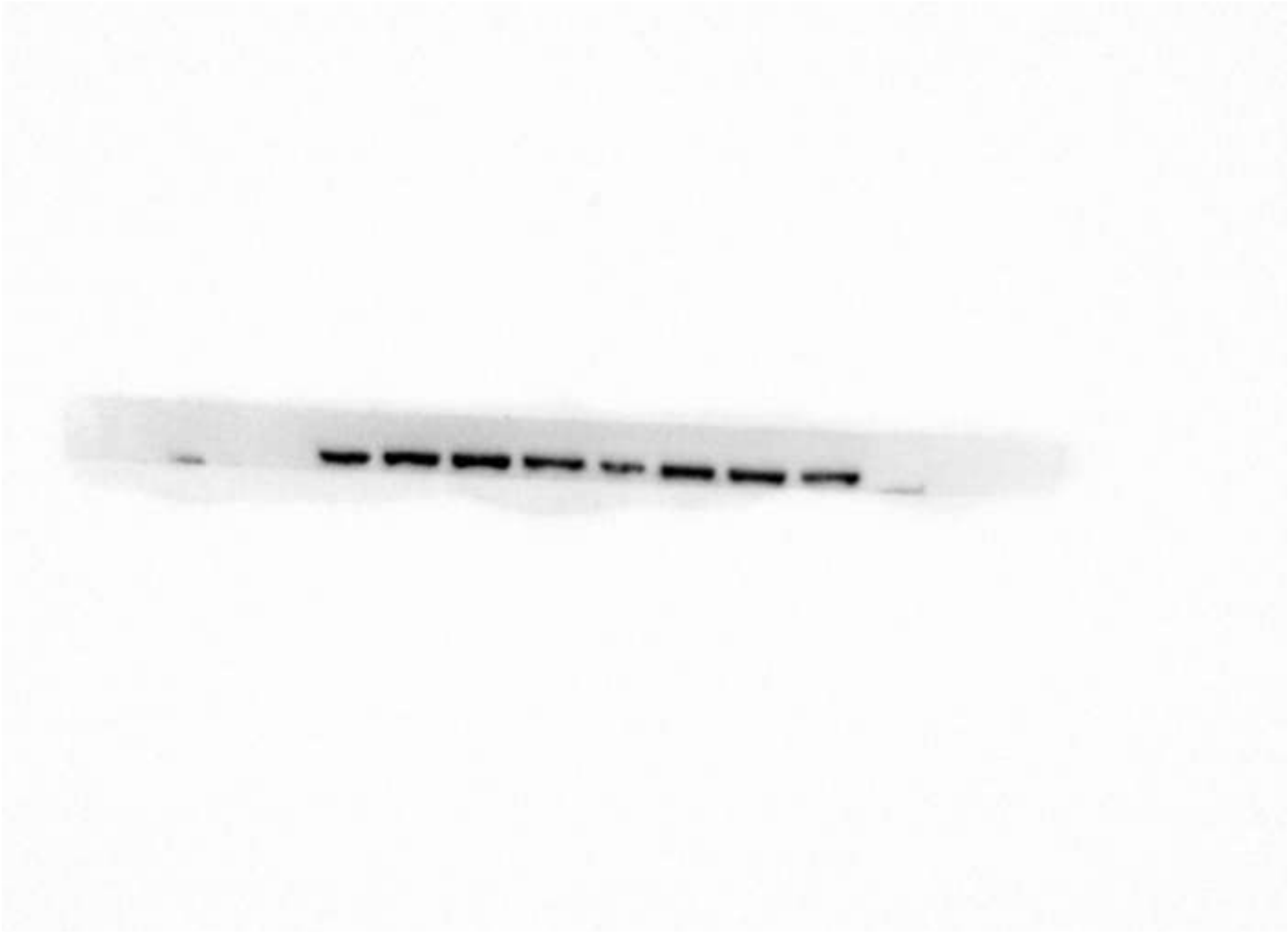

Full unedited gel for Figure 6D

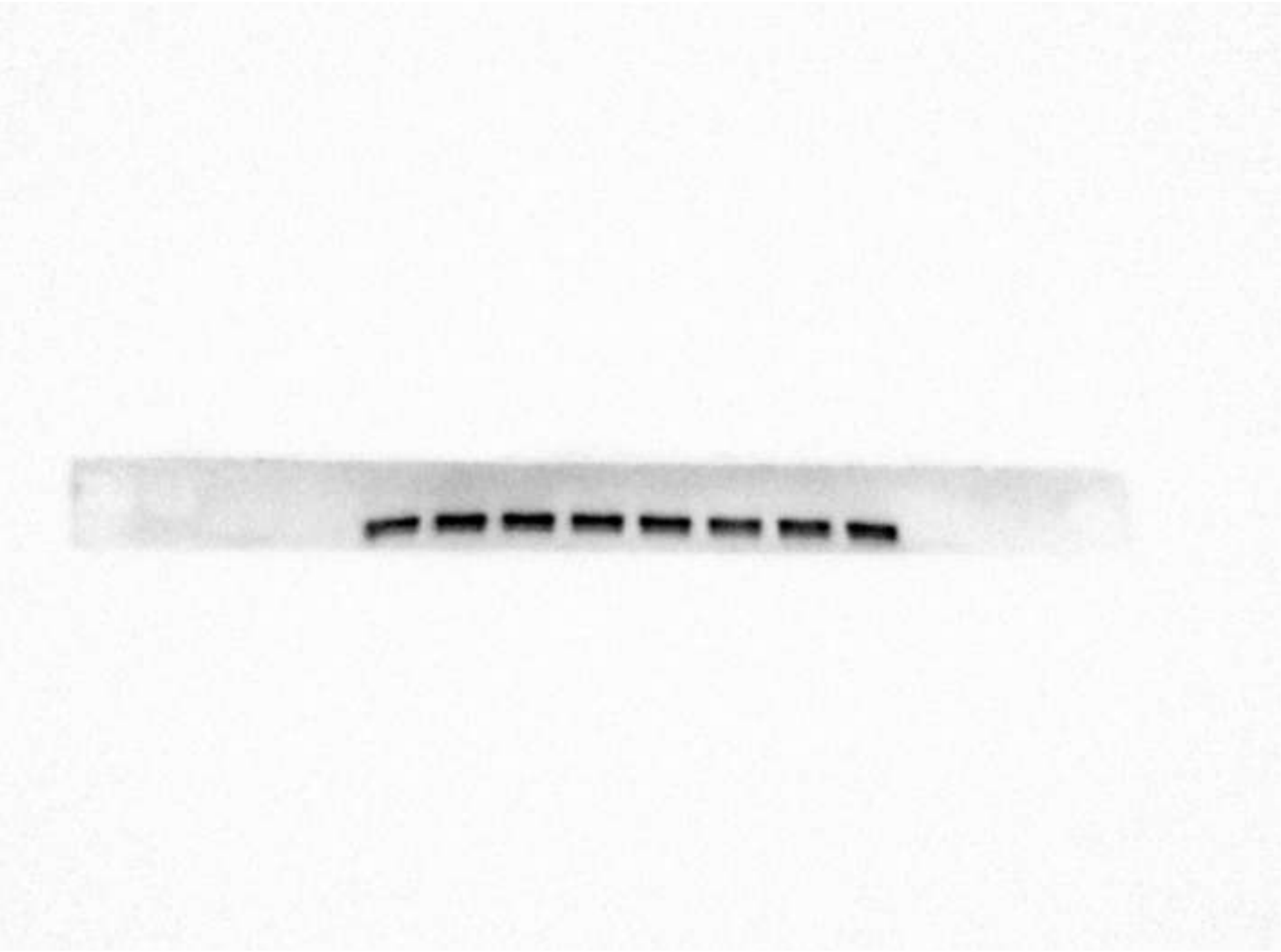

Full unedited gel for Figure 2E

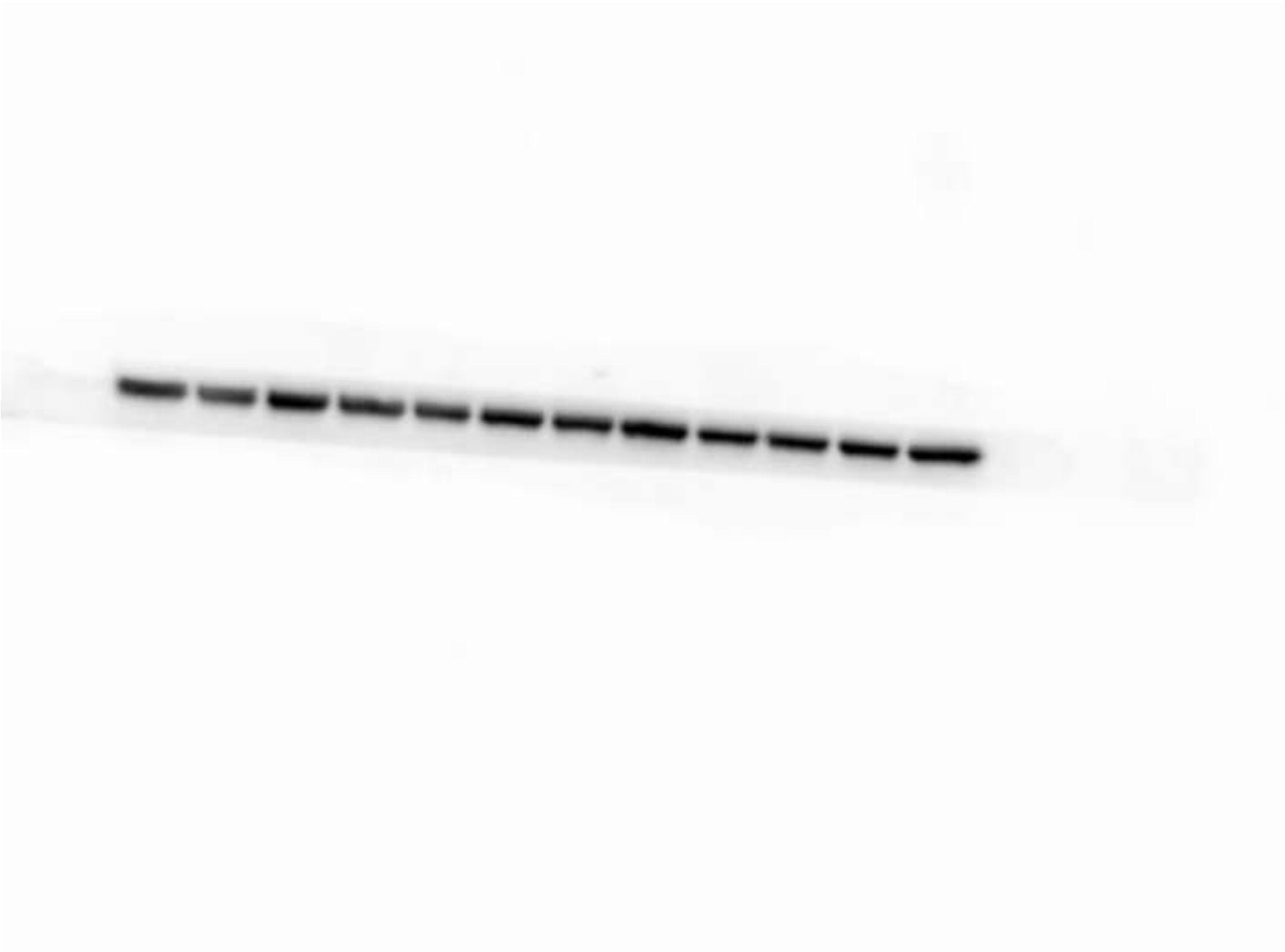

Full unedited gel for Figure 2D

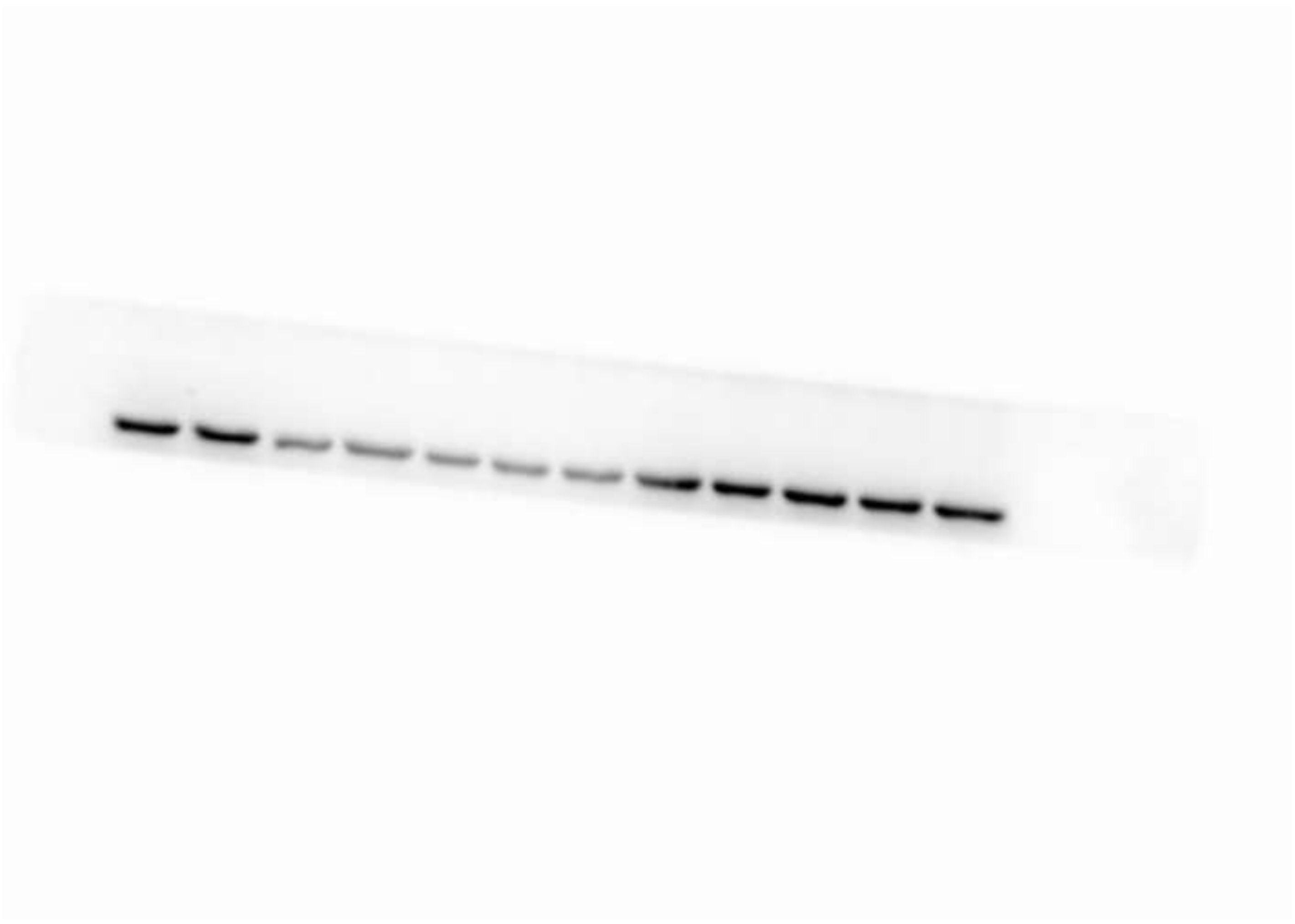

Full unedited gel for Figure 2E

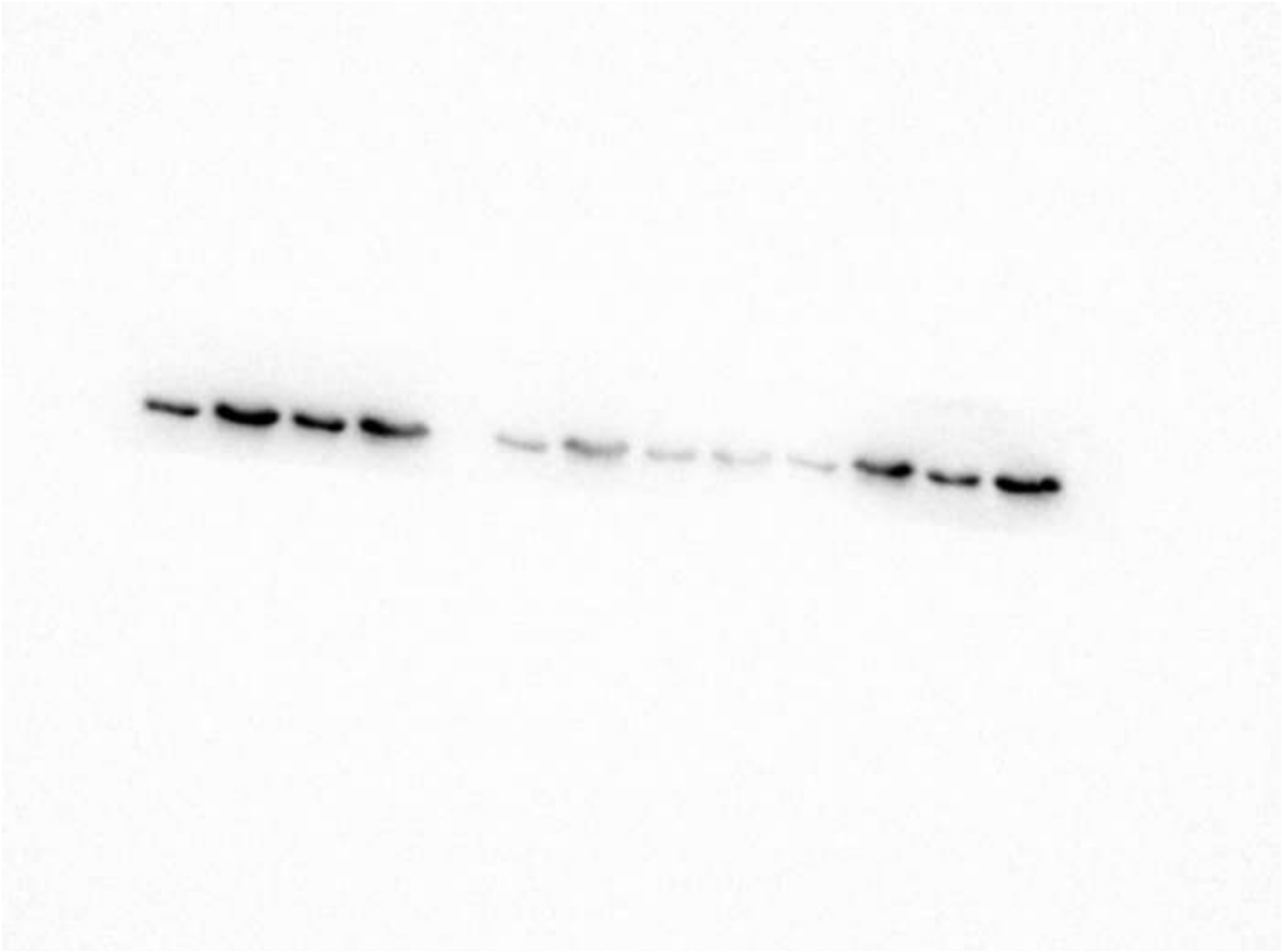

Full unedited gel for Figure 2D

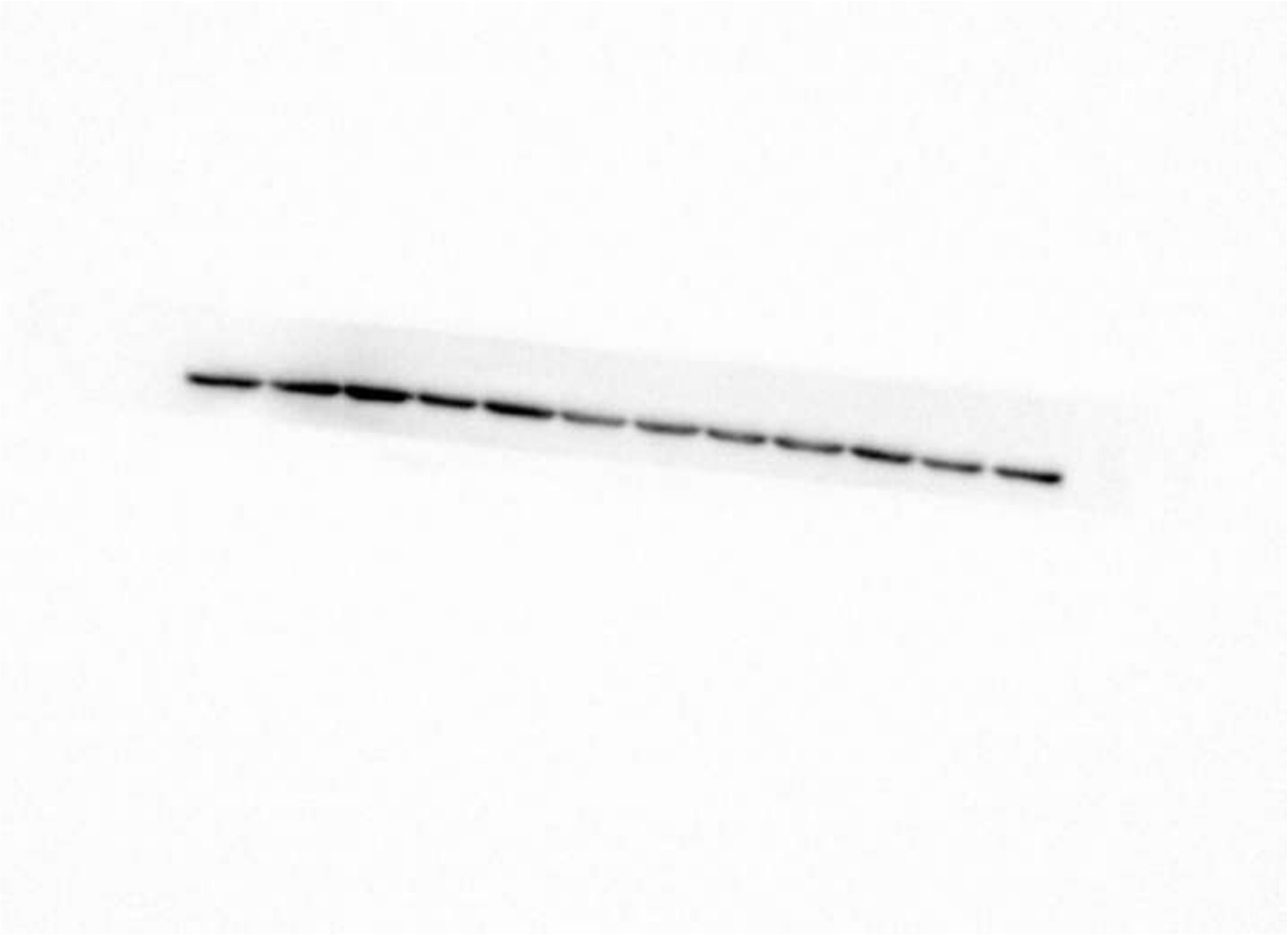

Full unedited gel for Figure 5D

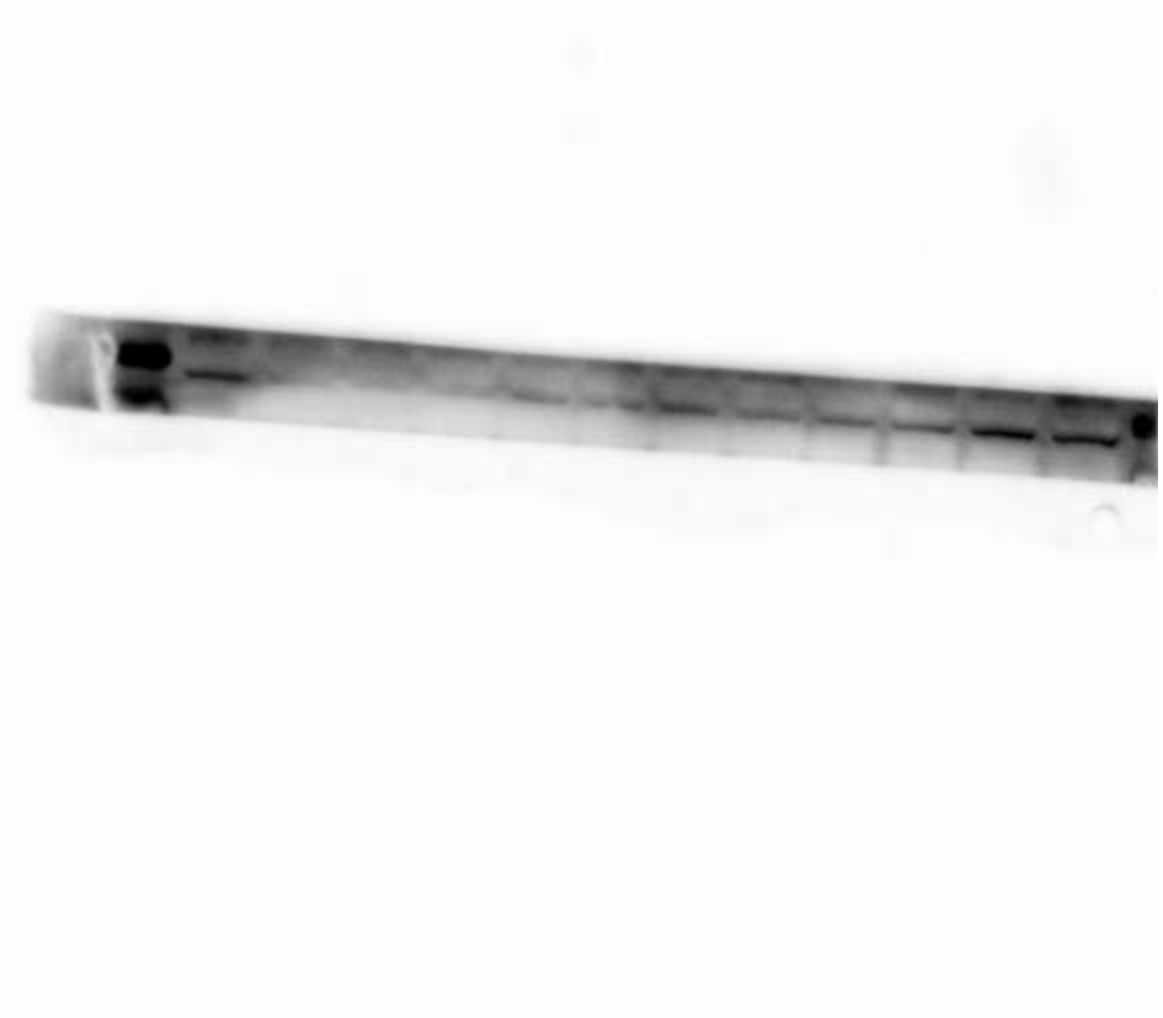

Full unedited gel for Figure 5D

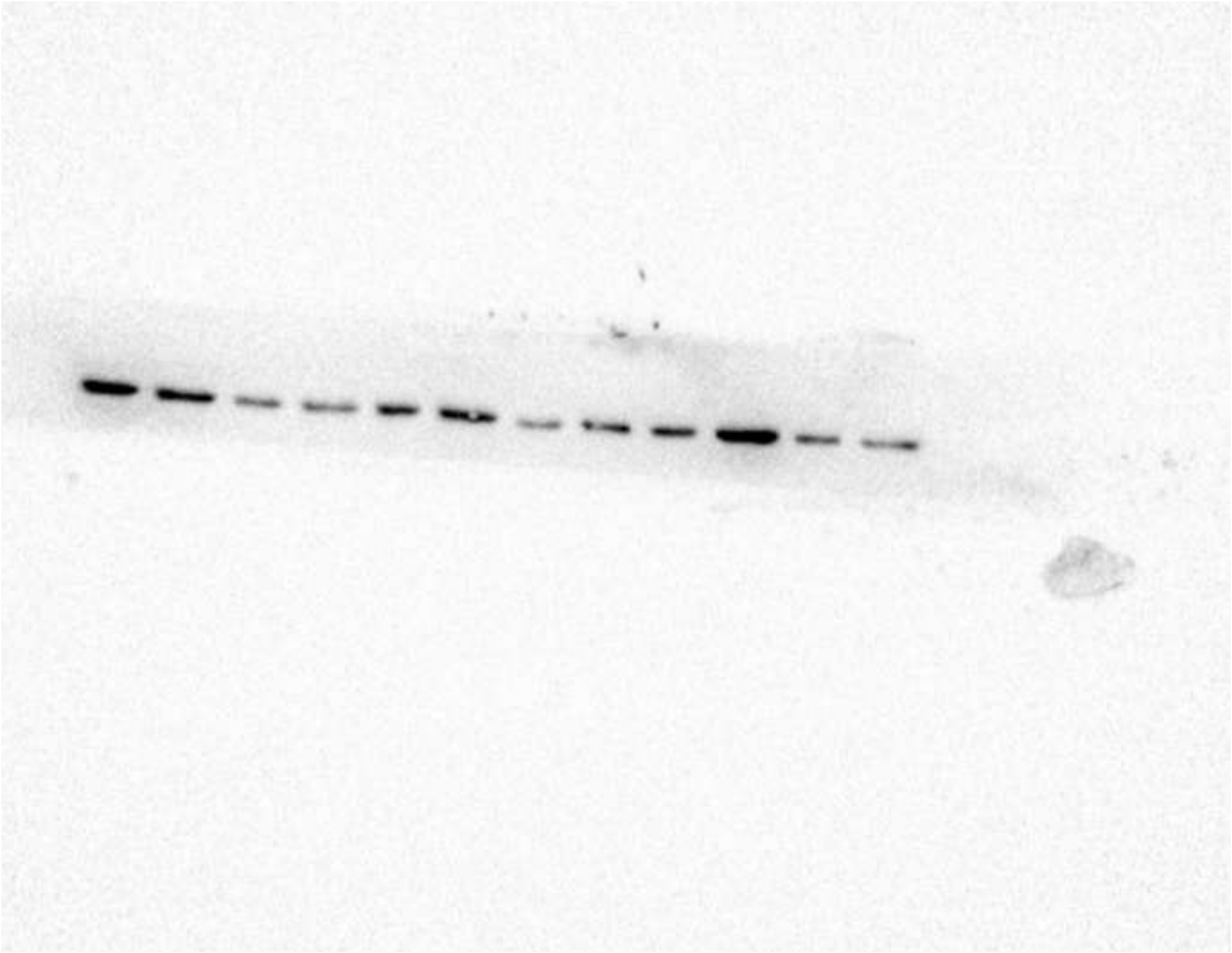

Full unedited gel for Figure 5D

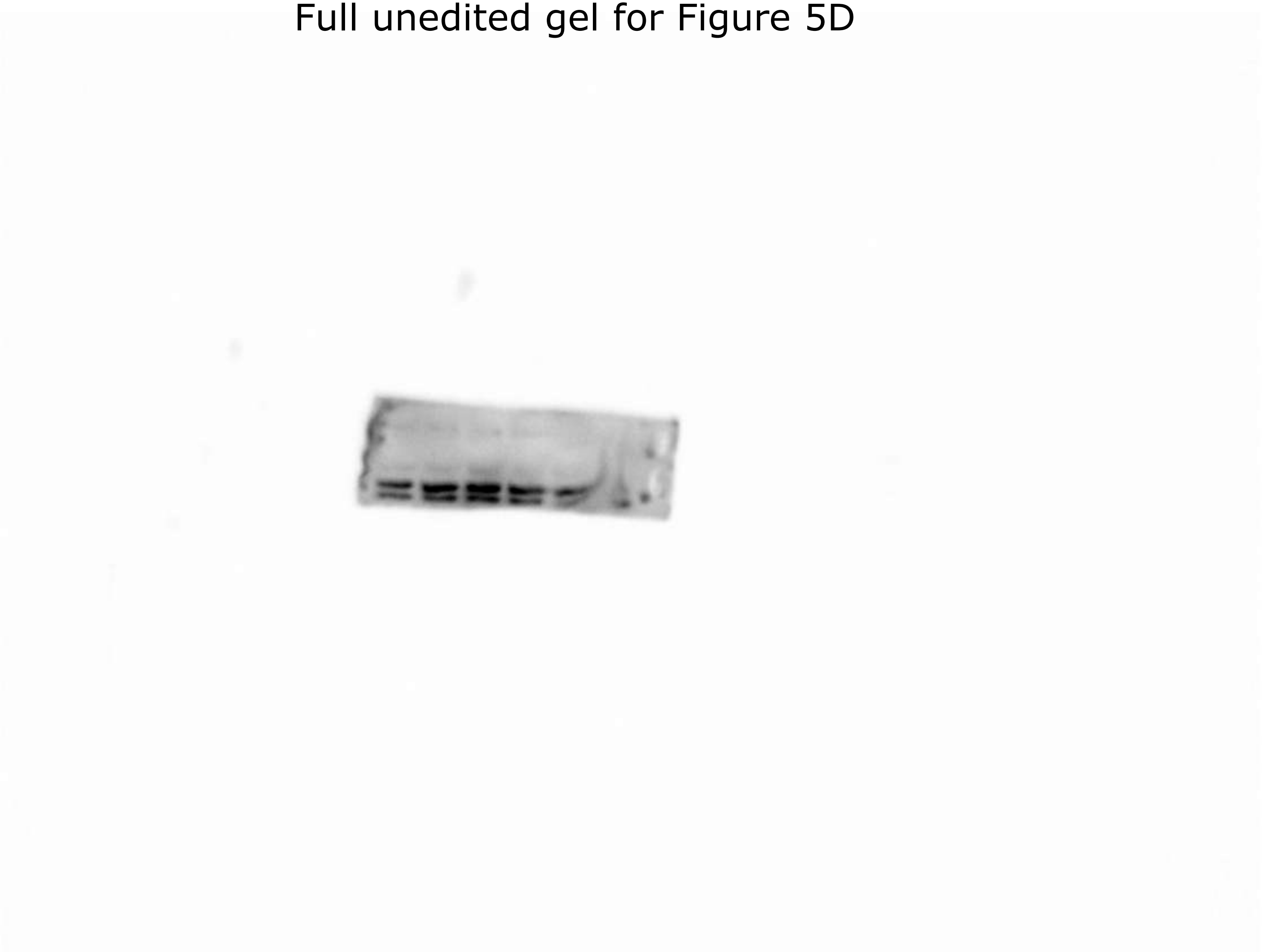

Full unedited gel for Figure 5D

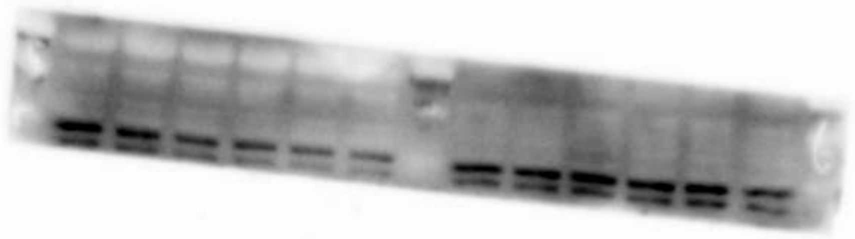

Full unedited gel for Figure 5D

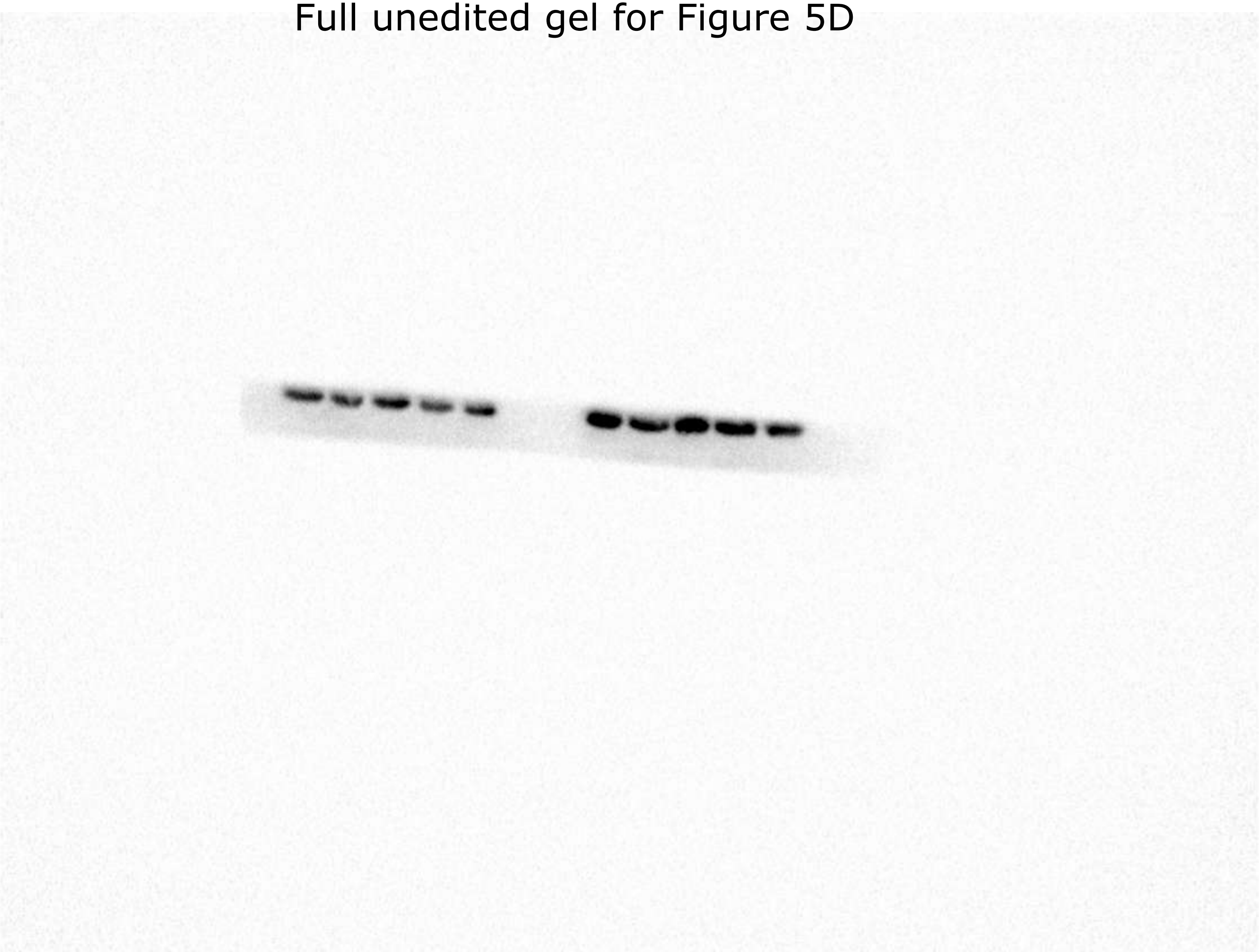

Full unedited gel for Figure 5D

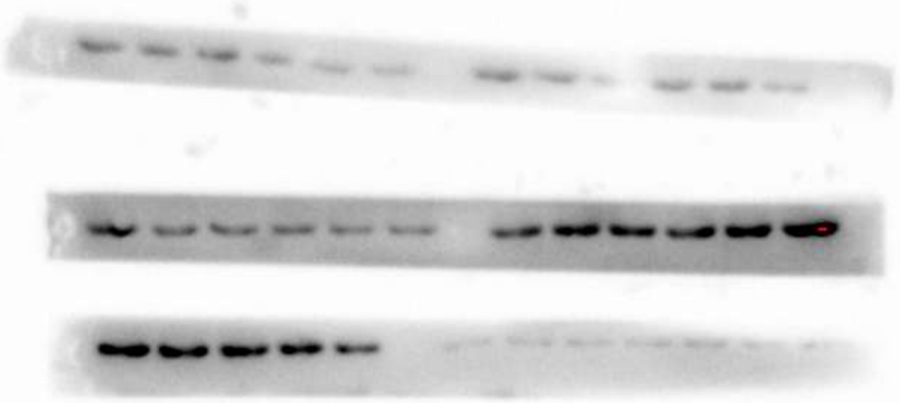

Full unedited gel for Figure 6C

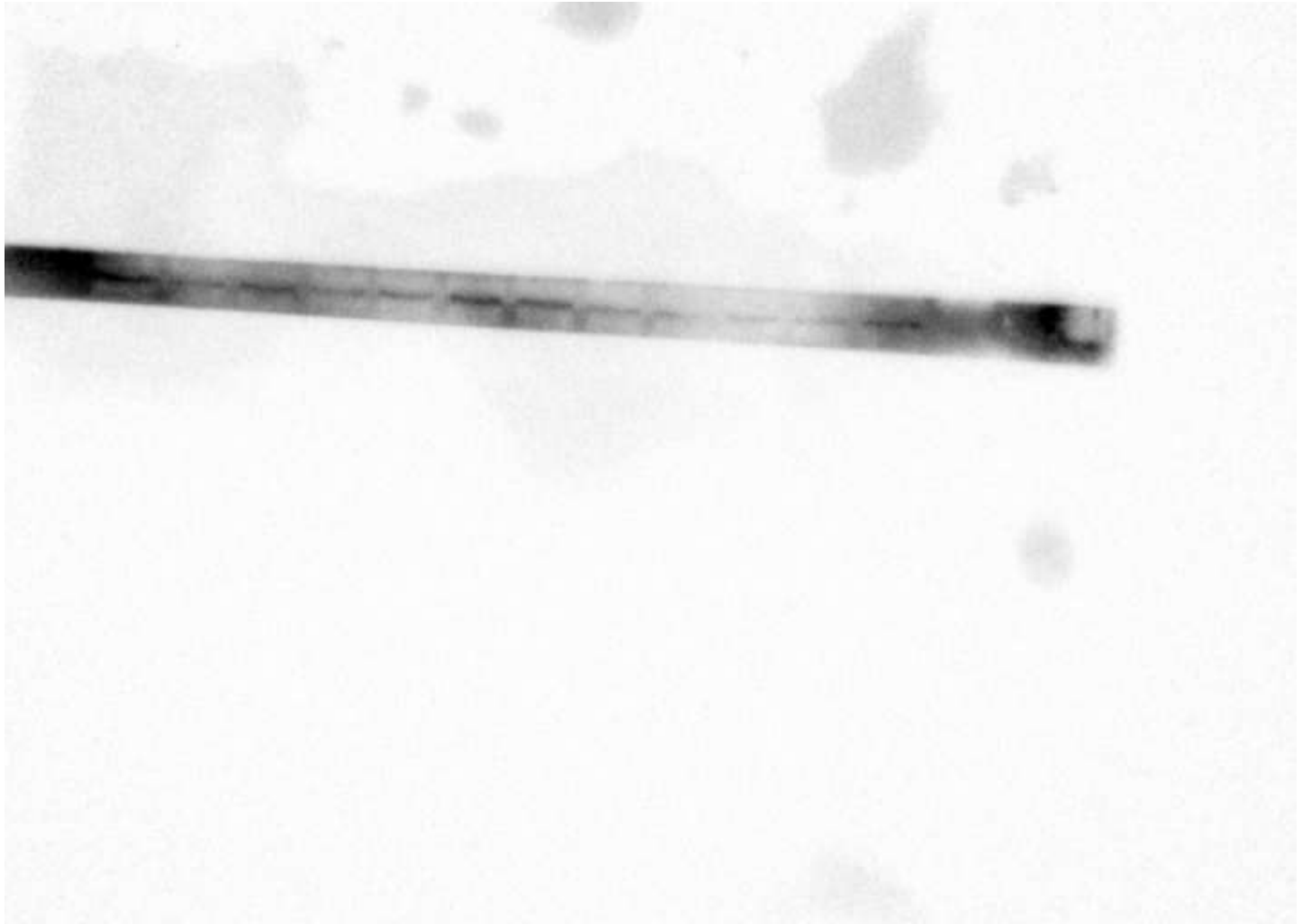

Supplement: Supplementary file 2 — Uncropped gels [file 41413_2022_186_MOESM2_ESM.pdf]
